# All-male hybrids of a tetrapod *Pelophylax esculentus* share its origin and genetics of maintenance

**DOI:** 10.1186/s13293-018-0172-z

**Published:** 2018-04-02

**Authors:** Marie Doležálková-Kaštánková, Nicolas B. M. Pruvost, Jörg Plötner, Heinz-Ulrich Reyer, Karel Janko, Lukáš Choleva

**Affiliations:** 1 Laboratory of Fish Genetics, Institute of Animal Physiology and Genetics, The Czech Academy of Sciences, 277 21 Liběchov, Czech Republic; 2 Department of Zoology, Faculty of Science, Charles University in Prague, 128 43 Praha 2, Czech Republic; 3 Institute of Evolutionary Biology and Environmental Studies, University of Zurich, Winterthurerstrasse 190, CH-8057 Zurich, Switzerland; 4 Museum für Naturkunde, Leibniz Institute for Evolution and Biodiversity Science, Invalidenstraße 43, 10115 Berlin, Germany; 5 Department of Biology and Ecology, Faculty of Science, University of Ostrava, Chittussiho 10, 710 00 Ostrava, Czech Republic

**Keywords:** *Pelophylax*, Water frog, Hemiclone, Hybridogenesis, Sexual parasites, Unisexual, All-male lineage

## Abstract

**Background:**

Sexual parasites offer unique insights into the reproduction of unisexual and sexual populations. Because unisexuality is almost exclusively linked to the female sex, most studies addressed host-parasite dynamics in populations where sperm-dependent females dominate. *Pelophylax* water frogs from Central Europe include hybrids of both sexes, collectively named *P. esculentus*. They live syntopically with their parental species *P. lessonae* and/or *P. ridibundus*. Some hybrid lineages consist of all males providing a chance to understand the origin and perpetuation of a host-parasite (egg-dependent) system compared to sperm-dependent parthenogenesis.

**Methods:**

We focused on *P. ridibundus*-*P. esculentus* populations where *P. ridibundus* of both sexes lives together with only diploid *P. esculentus* males. Based on 17 microsatellite markers and six allozyme loci, we analyzed (i) the variability of individual genomes, (ii) the reproductive mode(s) of all-male hybrids, and (iii) the genealogical relationships between the hybrid and parental genomes.

**Results:**

Our microsatellite data revealed that *P. esculentus* males bear Mendelian-inherited *ridibundus* genomes while the *lessonae* genome represents a single clone. Our data indicate that this clone did not recently originate from adjacent *P. lessonae* populations, suggesting an older in situ or ex situ origin.

**Conclusions:**

Our results confirm that also males can perpetuate over many generations as the unisexual lineage and successfully compete with *P. ridibundus* males for eggs provided by *P. ridibundus* females. Natural persistence of such sex-specific hybrid populations allows to studying the similarities and differences between male and female reproductive parasitism in many biological settings.

**Electronic supplementary material:**

The online version of this article (10.1186/s13293-018-0172-z) contains supplementary material, which is available to authorized users.

## Background

Unisexual animals are model systems for understanding the evolution and maintenance of sex and recombination despite the costs that may arise [1, 2]. Interspecific hybridization between diploid bisexual species is likely the only mechanism by which unisexuality originates in vertebrates [3]. In 46 described cases of squamate reptiles, 51 cases of fish, and 23 cases of amphibians, unisexuality is almost exclusively linked to the female sex [4–6]. Hybrid males are frequently inviable or sterile (e.g., [7]), but interestingly, a few cases of viable and likely fertile male vertebrates have been documented, e.g., in water frogs of the genus *Pelophylax* [8–10] and fish of the genera *Squalius* [11], *Misgurnus* [12, 13], and *Hypseleotris* [14]. An example from the *Squalius alburnoides* complex suggests that perpetuation of hybrid males in populations depends on highly complex reproductive mechanisms [11]. Here, hybrid females are sperm-dependent sexual parasites on the males of the host species which reproduces sexually, while hybrid males are rather a by-product of crosses between hybrid females and sexual males. A production of various types of gametes and presence of polyploid inividuals for male maintenance is rather requisite in this mating system. All-male hybridogenetic lineages were found in *Pelophylax* and *Hypseleotris*. It is not yet clear what their origin is and what enables the maintenance of male unisexuality in these systems.

Hybridogenesis, combining elements of both clonal and sexual reproduction, seems to be the most promising reproductive mode for the maintenance of male unisexuality as separate, sex-specific lineages [15]. Hybrids with hybridogenetic reproduction usually discard one complete parental genome from their germline prior to meiosis and clonally transmit the remaining one, generally the mother’s one [3, 16], except some modified cases [11, 17, 18]. Hybridity is restored in each generation through fertilization by the sexual species, whose genome has been eliminated in the germline of the hybrid. Hence, inheritance is typically half clonal and half sexual (Mendelian), referred to as hemiclonal [19].

The *Pelophylax esculentus* complex consists of two sexual species, the pool frog *Pelophylax lessonae* (genomic composition LL) and the the marsh frog *P. ridibundus* (RR), and their hybrid taxon, the edible frog *P. esculentus* (RL). In most parts of Western and Central Europe, *P. esculentus* lives in sympatry with *P. lessonae*, in what is known as the “L-E system” [20]. In this system, both male and female hybrids usually exclude the haploid *lessonae* (L) genome from their germline and transmit the *ridibundus* (R) genome to their gametes. Hybridity is restored in each generation by heterospecific crosses between *P. esculentus* and *P. lessonae*, the donor of the L genome. When a hybrid male mates with a LL or RL female, only female offspring originates. Thus, males originate from matings between hybridogenetic females and heterospecific sexual *P. lessonae* males producing both hybrid sexes [21].

Conversely, in the R-E system, most *P. esculentus* exclude the *ridibundus* genome, transmit their *lessonae* genome, and mate with *P. ridibundus* to perpetuate the hybrid lines [21–23]. Special cases are populations which consist of *P. ridibundus* and only *P. esculentus* males. Such populations have been reported from the Czech Republic [24], Germany [8, 9, 25], Hungary [26], and Poland [27–29]. In contrast to the L-E system, some *P. esculentus* males from this population type transmit only the *lessonae* genome, others only the *ridibundus* genome, and some males even produce both *ridibundus* and *lessonae* gametes [9, 23, 24, 30]. From artificial crossings between *P. ridibundus* females and *P. esculentus* males usually two genotypes originate: if the *ridibundus* (R) eggs are fertilized by sperm with an R genome, RR females originate, while the combination of R eggs an L sperm gives rise to only hybrid (LR) males [9, 10, 30, 31]. This offers a potential explanation for the existence and perpetuation of all-male lineages in natural populations [9]. So far, it is not known whether one or multiple hybridization events have led to the formation of all-male *P. esculentus*. Consequently, there has been, and still might be, the possibility for de novo formation of *P. esculentus* males via ongoing primary hybridizations between the two sexual species, thus leaving open the question of their stable persistence in natural populations.

This study was aimed to (1) explore the origin of all male lineages in populations of the R-E system, i.e., whether male unisexuality evolved from one or several hybridization events, and (2) to understand how all-male hybrids are able to persist without hybrid females in natural populations. In pursuing these goals, we sampled 249 water frogs from 16 populations along the upper Oder River. Based on 17 microsatellite markers, we investigated (a) the mode of asexual reproduction, (b) the genetic variability of clonally transmitted genomes, and (c) their genealogical relationships to homotypic genomes from adjacent populations.

## Methods

### Study species, sampling sites, and taxon assignment

**Fig. 1:**
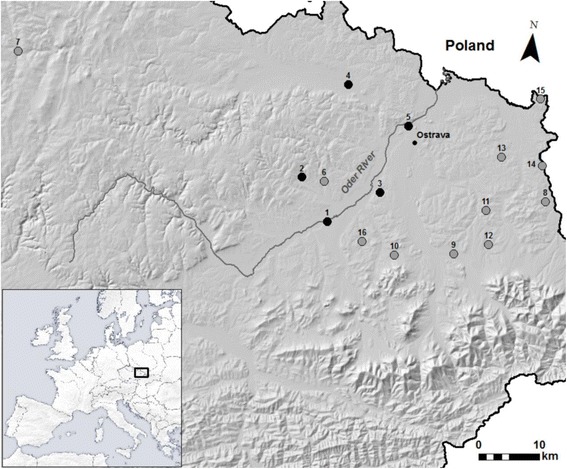
Map showing the investigated water frog populations in the upper Oder River. Numbers next to the dots correspond with numbers of localities given in Table 1. Dark gray dots refer to the R-E male system, light gray dots to the L-E system. The inset indicates the geographic location of the study area in Europe

All European water frogs were collected in the upper Oder River drainage (Central Europe; Czech Republic; Fig. 1, Table 1). In total, 249 individuals from all three *Pelophylax* taxa were caught with a hand net at 16 locations during years 2002 and 2008. *Pelophylax kurtmuelleri* from Greece, a sister species of European *P. ridibundus*, was used as an outgroup in a phylogenetic tree construction. Males were distinguished from females by the presence of vocal sacs and nuptial pads. Taxon affiliation was determined on the basis of external morphological characters [22, 32], and identification was later verified genetically with allozyme markers (see the “Protein electrophoresis” section). Tissue samples obtained from the finger tips or muscles were stored at − 20 °C for allozyme analyses and in 96% ethanol for DNA analyses.

**Table 1:** Origin and genetic data of 16 water frog populations from the upper Oder River valley. Given are population types, names, and coordinates for sampling sites, genotypes, and numbers of collected females (f)/males (m)/juveniles (-), types and numbers of the detected multilocus genotypes (MLG), and IDs of hemiclones. For more details, see Additional file 2: Table S2

No.*	Pop. type	Sampling site	Latitude Longitude	Genotype	No. of f/m/juv	MLG type	No. of MLGs	Hemiclone ID
1	R-E male	Albrechtičky	49.708056 18.098889	RR	8/9/-	RR	17	
				RL	-/12/-	R	12	
						L	1	OderL1
2		Bílovec	49.769722 18.032222	RR	1/-/-	RR	1	
				RL	-/2/-	R	2	
						L	1	OderL1
3		Darkovice	49.757778 18.213056	RR	3/5/-	RR	8	
				RL	-/4/-	R	4	
						L	1	OderL1
4		Dolní Benešov	49.912222 18.120000	RR	4/2/-	RR	6	
				RL	-/4/-	R	4	
						L	1	OderL1
5		Ostrava	49.858889 18.265278	RR	5/4/-	RR	9	
				RL	-/5/-	R	5	
						L	1	OderL1
6	L-E	Bravantice	49.766944 18.084444	LL	1/-/-	LL	1	
				RL	7/-/-	R	NA	
						L	7	
7		Břidličná	49.916389 17.359444	LL	-/4/-	LL	4	
8		Český Těšín	49.764444 18.591667	LL	3/7/-	LL	10	
				RL	5/-/-	R	NA	
						L	5	
9		Dobrá	49.676667 18.392222	LL	7/22/2	LL	31	
10		Důl Staříč	49.667222 18.258611	LL	-/2/-	LL	2	
				RL	13/-/-	R	NA	
						L	13	
11		Horní Bludovice	49.744444 18.457500	LL	6/15/-	LL	21	
				RL	2/2/-	R	NA	
						L	4	
12		Horní Domaslavice	49.694722 18.470000	LL	3/4/-	LL	7	
				RL	7/1/-	R	NA	
						L	8	
13		Karviná-Doly	49.825000 18.483056	LL	-/2/-	LL	2	
				RL	10/3/-	R	NA	
						L	13	
14		Louky	49.817222 18.576944	LL	1/-/-	LL	1	
				RL	10/4/-	R	NA	
						L	14	
15		Prstná	49.915000 18.560556	LL	3/5/-	LL	8	
				RL	3/-/-	R	NA	
						L	3	
16		Trnávka	49.683333 18.181389	LL	8/10/3	LL	21	
				RR	-/-/1	RR	1	
				RL	4/-/-	R	NA	
						L	4	
OG	KK	Greece	39.873166 22.732813	KK	1/-/-	KK	1	

*R-E male P. ridibundus-P. esculentus* male populations, *L-E P. lessonae-P. esculentus* populations, *OG* used as an outgroup, *RR P. ridibundus*, *RL P. esculentus*, *LL P. lessonae*, *KK P. kurtmuelleri*, *NA* not analyzed

*The localities are numbered according to Fig. 1

### Protein electrophoresis

For genotype determination, six allozyme loci previously identified to be diagnostic for *Pelophylax* taxa were used [20, 33]. Approximately 1 g of skeletal muscle (or gonads) was homogenized on crushed ice for 20 s in an equal volume of Tris NaCl extraction buffer (pH 8.5; [34]) using an Ultra-Turrax homogenizator (IKA-WERK). The homogenate was then centrifuged at 13,500*g* at 4 °C for 20 min. Enzymes obtained from these tissues, namely aspartate aminotransferase (*Aat*; EC 2.6.1.1), glucose-6-phosphate isomerase (*Gpi*; *EC* 5.3.1.9), glycerol-3-phosphate dehydrogenase (*G3pdh*; EC 1.1.1.8), l-lactate dehydrogenase (*Ldh-1*; EC 1.1.1.27), phosphoglucomutase (*Pgm-2*; EC 5.4.2.2), and phosphogluconate dehydrogenase (*6-Pgd*; EC 1.1.1.44), were analyzed by horizontal potato starch gel electrophoresis [20, 34]. Subsequently, gels were cut into three 2-mm-thick slices and stained with appropriate allozyme chromogenic detection methods [35–37]. Stained gel slices were photographed and the agar layers were transferred to filter paper, then dried and stored as part of the protocol. The visualized allele products were designated from “*a*” fastest to “*e*” slowest according to their mobility. Samples which revealed unclear patterns were reprocessed.

### DNA extraction, microsatellite genotyping

Genomic DNA was extracted using a NucleoSpin commercial kit (Macherey-Nagel GmbH and Co.) with the epMotion 5075 automated pipetting system (Eppendorf). We amplified 17 microsatellite loci: Ga1a19, Re2caga3, Re1Caga10, RICA1b6 [38], RlCA1b5, RlCA5, RlCA18 [39], RlCA2a34, GA1a23, Rrid169A, Rrid059A, RlCA1a27, Rrid135A [40], Res16, Res20, Res22 [41], and Rrid013A [39, 42], using the redesigned primer sets described in Hermaniuk et al. [43]. We followed Pruvost et al. [31] for species-specific characterization of the aforementioned markers. Polymerase chain reaction (PCR) was performed according to Christiansen and Reyer [40]. Fragment lengths were determined using an ABI 3730 Avant capillary sequencer (Applied Biosystems, Zug, Switzerland) and an internal size standard (GeneScan-500 LIZ); alleles were scored with GeneMapper v. 3.7 (Applied Biosystems, Zug, Switzerland).

### Preparing datasets used for all analyses

Due to the presence of null alleles, raw microsatellite genotypes of *P. lessonae* and *P. ridibundus* were checked for potential genotyping errors with Micro-Checker version 2.2.3 [44]. This method estimates frequencies of null alleles with the Brookfield 2 null allele estimator, which treats nonamplifications as data and regards them as null homozygotes when calculating null allele frequencies [45]. In each population, Micro-Checker tested every locus for departure from the Hardy-Weinberg (*HW*) genotypic equilibrium. We found one locus (Res20) in *P. lessonae* and four loci (Res16, Rrid069A, RICA5, Re1Caga10) in *P. ridibundus* with a potential presence of null alleles, and therefore applied a correction for null alleles at these loci following Wagner et al. [46]. The after-correction analysis in Micro-Checker did not detect any locus with a presence of null alleles. The software MSA v. 4.05 [47] was used to determine summary statistics like mean microsatellite allele numbers (*AN*), observed heterozygosities (*H*_OBS_), and expected heterozygosities (*H*_EXP_) within populations.

Based on the definition of hybridogenetic reproduction, diploid hybrid water frogs from Central Europe transmit one haploid chromosome set clonally to gametes (therefore termed a “hemiclone” [19]), whereas the other set is discarded and regained for the next generation by mating with an individual of the sexual parental species. As our aim was to test the origin of a particular hemiclone in this vertebrate species, we analyzed sexual and clonal genomes of hybrid individuals separately. First, we sorted *lessonae* and *ridibundus* genomes according to the allele species specificity known from the literature [31]. Then, the correctness of allele separation was tested visually assuming that one allele per locus was received from a sexual mate, and therefore such allele has to be present (and always was in our study) in the gene pool of a sympatric sexual population. Our approach of separating sexually and clonally inherited alleles in hybrid genomes was 100% successful.

While for hybrids from R-E system populations both *ridibundus* and *lessonae* genomes were included in our analyses, we used only *lessonae* genomes of hybrids from the L-E system populations because in this system we were not interested in the origin of *ridibundus* genomes. We determined a hemiclone by a multilocus genotype (MLG), defined by the identical combination of alleles found in our microsatellite analyses. A minimum of three samples exhibiting the same allele composition was a clear indication that the genome was inherited clonally (*P*_SEX_ value mentioned below) and did not originate from a sexual donor [48]. As most statistical programs dealing with microsatellite data—including GenAlEx and Populations used in this study—are not designed to compare haploid and diploid data, we transformed the haploid genotypes to diploid forms adding a second allele as missing data. This was done for *lessonae* and *ridibundus* genomes of *P. esculentus* (MLG’s) separately. To diploidize these haploid genotypes for GeneClone, we simply doubled *lessonae* and *ridibundus* genomes. Detailed information about the programs used are given below.

### Flow cytometry

In order to determine ploidy levels, all individuals were analyzed by flow cytometry using blood samples following the standardized methodology [49]. A drop of blood was added into 70% ethanol and immediately shaken to prevent clotting. Chicken blood was used as a reference standard for cell size measurement. Relative nuclear DNA content was measured with DAPI fluorochrome using the Cystain two Step High Resolution DNA Staining commercial kit (Partec GmbH, Münster, Germany). Fluorescence intensity of 5000 stained nuclei was measured with a Partec PAII flow cytometer at a speed of 0.5 μl/s. Flow cytometric histograms were evaluated using FloMax 2.52 (Partec GmbH, Münster, Germany).

### Statistical analysis of microsatellite data

In order to determine the origin of the all-male *P. esculentus* lineages, we analyzed genetic relationships between hybrids and their parental species on the basis of allele frequencies of up to 17 microsatellite loci using Structure v. 2.3.1 [50], GenAlEx v. 6.5 [51], Populations v. 1.2.32 [52], and GeneClone v.2.0 [53].

#### Hybrid origin

The origin of *P. esculentus* was analyzed with a model-based clustering method as implemented in Structure to infer population structure based on genotypic data consisting of unlinked markers. Structure analyses included datasets of 177 individuals (27 hybrid males from the R-E populations, 108 *P. lessonae* and 42 *P. ridibundus*, Additional file 1: Table S1)*.* The analysis was carried out using a burn-in period of 10,000 iterations followed by 100,000 Markov Chain Monte Carlo (MCMC) repeats. The probability of the used admixture model was tested for clusters *K* = 1 − 10. The most probable number of *K* populations was estimated using log-likelihood ln *P*(*D*) according to Evanno et al. [54], with Structure Harvester [55].

#### Hemiclonal reproduction

In order to distinguish whether individual genomes are of sexual or clonal origin, we estimated *P*_SEX_ values using the program GeneClone. When the same genotype is detected more than once, *P*_SEX_ expresses the probability that this MLG has been derived from distinct reproductive events [56]. We applied the *P*_SEX_ probability taking into account the *F*_IS_ estimated of the dataset [57]. *F*_IS_ was also estimated on the basis of the round-robin method, and further used to estimate a corrected *P*_GEN_, calculated as the unique MLG probability [56], which in turn provides a better estimate of the probability of clonal identity *P*_SEX_ [53]. Considering that the presence of missing data precludes the use of *P*_SEX_ in the program, we considered 10 loci from which both genomes were amplified (Ga1a19, Re1Caga10, RICA1b6, RlCA1b5, RlCA5, RlCA2a34, GA1a23, Rrid059A, Res16, Rrid013A), i.e., MLGs with missing alleles were eliminated (Additional file 2: Table S2). Therefore, GeneClone analyses were run on 143 MLGs which included 21 hybrid males from the R-E populations, 85 *P. lessonae* and 22 *P. ridibundus* individuals (Additional file 1: Table S1)*.*

#### Hemiclones and their genetic relatedness

To sort out MLGs of 177 individuals (27 hybrid males from the R-E populations, 108 *P. lessonae*, 42 *P. ridibundus*), we used multilocus matches analysis as implemented in the program GenAlEx. In order to estimate the genetic relatedness between individual genomes, we compared allele frequencies, heterozygosity, and polymorphism estimates with the program GenAlEx*.* A centred principal component analysis (PCA) was applied to examine clustering of individuals based on total variation of microsatellite allele frequencies without scaling of alleles. For the PCA, we converted a list of 275 MLG’s from 17 loci (Additional file 2: Table S2) into a genetic distance matrix (covariance matrix with data standardization) and then used standard PCA to visualize the results. The PCA dataset included also the *lessonae* genome of 71 male and female hybrids from the L-E system (Additional file 1: Table S1).

UPGMA trees (unweighted pair group method with arithmetic mean, [58]) were constructed using Nei’s *DA* distance [59] calculated on the basis of 205 MLGs (Additional file 1: Table S1) with the program Populations which works with datasets containing rather lesser amount of missing data. Therefore, all loci that amplified only in one parental species (Re2caga3, Res22, Rrid169A, Res20, RICA1a27, RICA18) and a locus Rrid135A which amplified only in some individuals were excluded from the analysis. Support for internal branches was evaluated with 7000 replicates. Distinct genetic groups of individual genomes were identified with PCA on the basis of Nei’s *DA*.

## Results

### Taxon determination

In total, we identified 108 individuals of *P. lessonae*, 42 individuals of *P. ridibundus*, and 98 individuals of *P. esculentus* using six diagnostic allozyme loci (Additional file 3: Table S3) and 17 microsatellite loci (Additional file 2: Table S2). In *P. lessonae*, there were four monomorphic (*Aat*, *Gpi*, *Pgm*, *G3pdh*) and two polymorphic allozyme loci (*Ldh-1*, *6-Pgd*); in *P. ridibundus*, two monomorphic (*Aat*, *G3pdh*) and four polymorphic loci (*Ldh-1*, *Gpi*, *6-Pgd*, *Pgm*) were found. In *P. esculentus*, all six loci were polymorphic. The allelic products of three loci (*G3pdh*, *Pgm*, *Gpi*) displayed an atypical expression in 12 individuals. Here, four *P. ridibundus* possessed alleles characteristic of the *lessonae* genome in *G3pdh* and *Pgm*, one *P. lessonae* possessed alleles characteristic of the *ridibundus* genome in *Pgm,* and seven *P. esculentus* possessed either two *lessonae* or two *ridibundus* specific alleles in both *Gpi* and *Pgm* (Additional file 3: Table S3). Whether the shared alleles represent introgressions caused by crosses between individuals of the parental species and hybrids or ancestral polymorphisms is not clear.

### Genetic diversity

Overall, all 17 microsatellite loci were polymorphic among 249 *Pelophylax* individuals (Additional file 4: Table S4). The loci Re2caga3, Res22, and Rrid169A amplified only in the *P. ridibundus* genome while the loci Res20, RICA1a27, and RICA18 amplified only in *P. lessonae* genome. Two loci (RICA5, Ga1a23) that are assumed to be specific for *P. lessonae* [39, 40], and one locus (Rrid135A), suggested to be specific for *P. ridibundus* [40], amplified in both *P. lessonae* and *P. ridibundus* samples.

A total of 187 alleles were detected, with 6 - 17 alleles per polymorphic locus (Additional file 2: Table S2, Additional file 4: Table S4 and Additional file 5: Table S5). The allele called 83 in RICA1b6 was marked as non-specific because it was amplified in the genomes of both *P. lessonae* (12 individuals) and *P. ridibundus* (four individuals). The presence of one or two alleles per locus (Additional file 2: Table S2) supported the flow cytometric data that all individuals were diploid. In addition, allele-dosage effects as indications of polyploidy were not observed.

### Analyses of population structure using microsatellite data

**Fig. 2:**
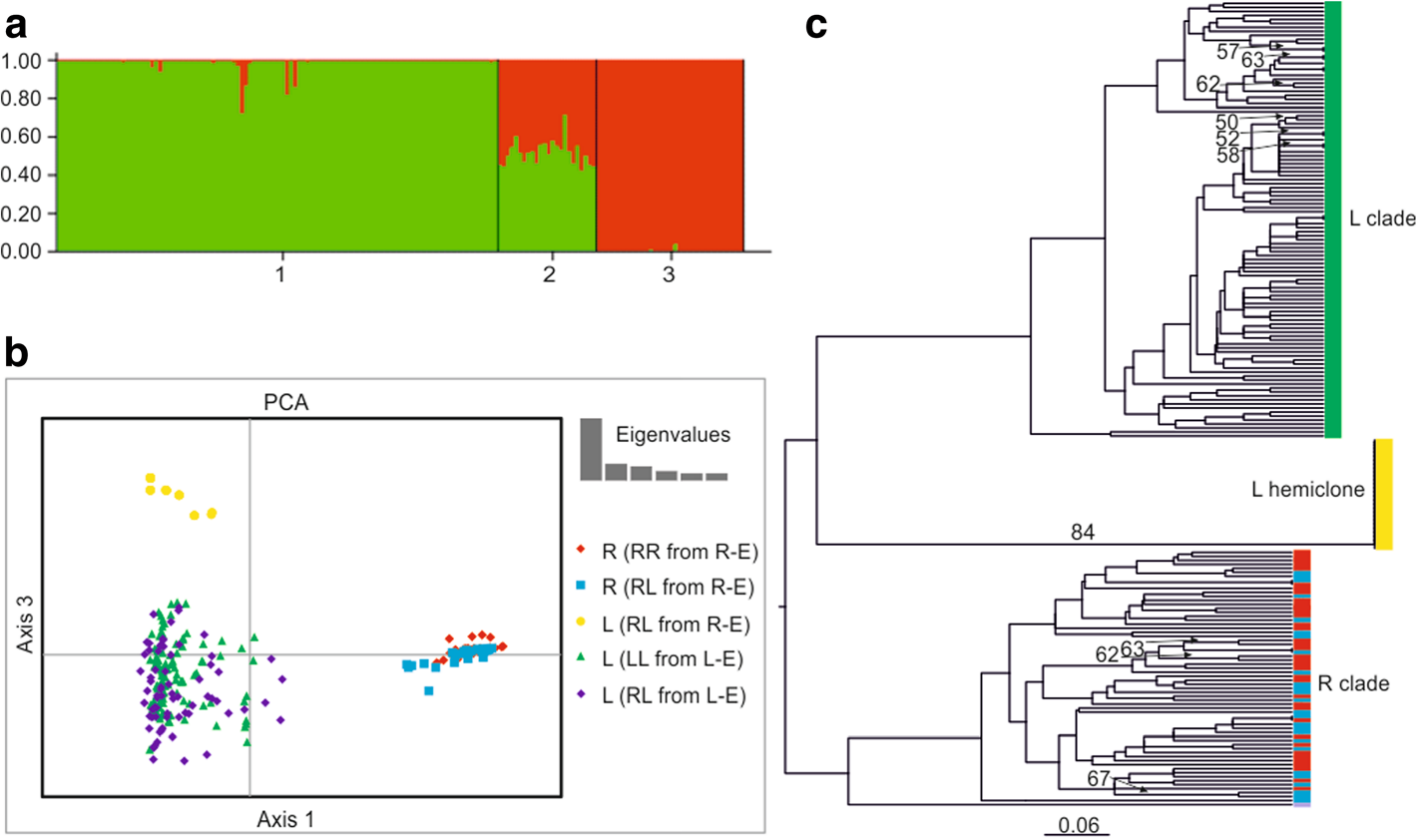
Cluster analyses performed on MLGs of *Pelophylax* individuals based on microsatellite loci. **a** Bar plot of 17 microsatellite loci from Bayesian cluster analysis performed in Structure (*K* = 2). Each vertical line represents one individual, each color represents the species specificity of alleles to one of the parental genomes (green = *P. lessonae* genome, red = *P. ridibundus* genome), and each cluster represents a different genotype (cluster 1 = LL, cluster 2 = RL, cluster 3 = RR). **b** Principal component analysis (PCA) of 17 microsatellite loci performed in GenAlEx. Each point represents an individual MLG, each color and symbol a group of related MLGs (according to allele sharing). Group red diamonds—R (RR from R-E) represents *P. ridibundus* from R-E system; group blue squares—R (RL from R-E) represents *ridibundus* genomes of *P. esculentus* from the R-E system; group yellow circles—L (RL from R-E) represents *lessonae* genomes of *P. esculentus* from the R-E system; group green triangles—L (LL from L-E) represents *P. lessonae* from the L-E system; group violet diamonds—L (RL from L-E) represents *lessonae* genomes of *P. esculentus* from the L-E system. Inset screenshot shows the eigenvalues for each axis as principle components of the analysis. **c** UPGMA tree computed with the program Populations using DA distances which were calculated on the basis of 10 microsatellite loci. Numbers on branches (with or without arrows) indicate bootstrap values > 50%. Each terminal unit represents one individual: green color—L clade represents *P. lessonae* from the L-E system; yellow color—L hemiclone represents *lessonae* genomes of *P. esculentus* from the R-E system; red color—R clade represents *P. ridibundus* from the R-E system; blue color—R clade represents *ridibundus* genomes of *P. esculentus* from the R-E system; violet color—*P. kurtmuelleri* individual. Detailed information is given in Additional file 6: Figure S1

Likelihood values provided by Structure converged during the runs, and results did not notably change between replicates. Evanno et al.’s [54] method implemented in Structure Harvester indicated *K* = 2 as the most probable number of genetic clusters for the dataset. Three types of diploid individuals were found, i.e., those with a *q* value ranging between 0.004–0.009 and 0.991–0.996, respectively, representing sexual *P. lessonae* or *P. ridibundus*, and those with intermediate *q* values (0.440–0.560) representing hybrid *P. esculentus*, which is in accordance with results from the determination based on diagnostic markers (Fig. 2a).

The PCA identified three groups of microsatellite MLGs, the first two principle components accounted for 62.15% (axis 1) and 11.47% (axis 3) of genetic variation, respectively (Fig. 2b). Cluster 1 (green and dark blue symbols) grouped *P. lessonae* and *lessonae-*specific MLGs of *P. esculentus* sampled in L-E system populations*.* Cluster 2 (yellow symbols) included *lessonae*-specific MLGs obtained from *P. esculentus* in R-E populations while cluster 3 (red and blue symbols) grouped *P. ridibundus* and *ridibundus*-specific MLGs of *P. esculentus* from R-E populations.

Genealogical analyses made with the program Populations revealed two distinct clusters of *lessonae*-specific MLGs. One is characteristic of *lessonae*-specific MLGs found in *P. esculentus* from R-E populations; the second comprises genotypes of *P. lessonae* individuals (Fig. 2c and Additional file 6: Figure S1). Contrary to the previous pattern, *ridibundus*-specific MLGs found in *P. esculentus* from R-E populations did not represent a separate lineage but instead clustered with *P. ridibundus*.

#### Identification of MLGs and hemiclones

**Fig. 3:**
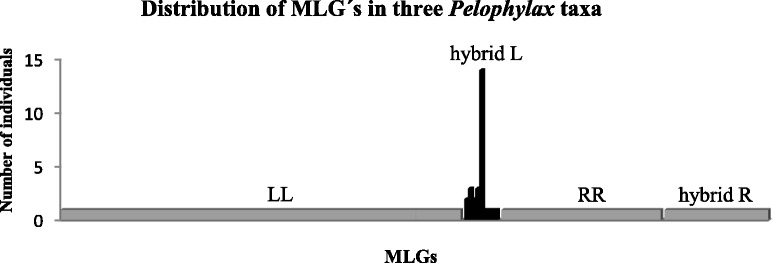
Distribution of 185 microsatellite multilocus genotypes (MLGs) in three *Pelophylax* taxa generated in GenAlEx (LL, *P. lessonae* from the L-E system; hybrid L, *lessonae* genome of *P. esculentus* from the R-E system; hybrid R, *ridibundus* genome of *P. esculentus* from the R-E system; RR, *P. ridibundus* from the R-E system)

In the dataset which contained missing data for some loci, GenAlEx estimated a total of 185 MLGs among all *Pelophylax* individuals investigated. Among 150 *P. lessonae* and *P. ridibundus* individuals, the program distinguished 150 MLGs. Considering the 27 *P. esculentus* from R-E male populations, *ridibundus* genomes were represented by 27 MLGs, whereas the *lessonae* genomes exhibited only eight MLGs (Fig. 3, Additional file 7: Table S6). One *lessonae*-specific MLG was present in 14 *lessonae* genomes of *P. esculentus*, while the remaining seven MLGs were discovered in one to three *lessonae* genomes. In contrast to MLGs obtained from *ridibundus* genomes, all *lessonae*-specific MLGs shared the same alleles; their differences are only caused by missing data for some loci (Additional file 7: Table S6). Therefore, we consider the eight *lessonae*-specific MLGs detected in the hybrid males of the R-E populations as a single MLG. This MLG is further suggested to represent a clone (or a hemiclone from an individual level) designated as ODERL1.

Considering only MLGs with complete allelic data, GenClone generated 122 MLGs among 143 genotypes. Twenty-one *lessonae* genomes, all originating from *P. esculentus* of R-E male populations, were represented by only a single MLG. The *P*_SEX_ value of this MLG ranged from 0.15 to 1.50E−41, indicating clonal inheritance of ODERL1.

## Discussion

### Detection of all-male *P. esculentus* lineage

The upper Oder River valley is mainly inhabited by *P. ridibundus* of both sexes and only males of *P. esculentus* (R-E system; populations 1-5, Table 1), whereas populations of the L-E system (typically represented by *P. lessonae* and *P. esculentus* of both sexes) live outside the valley. Since 2001, no hybrid female was detected among 377 hybrid individuals in localities of the R-E system (Table 1 and unpublished data), which makes this region a newly discovered area for the coexistence of bi-sexual *P. ridibundus* with diploid all-male *P. esculentus* hybrids. Together with the occurrence of similar populations downstream the Oder River drainage basin [8, 9, 28, 60], this points to Central Europe as the area of origin of R-E male populations.

### *P. esculentus* males are active in maintaining their own all-male hybrid lineage

Hybrid males are usually infertile [61] and therefore considered as a by-product of hybridizing sexual species or a sexual male species with a unisexual female species [7, 62]. In the L-E system of Western Europe, *P. esculentus* males usually result only from crosses between *P. esculentus* females and heterogametic *P. lessonae* males. Backcrosses between such hybrid males and *P. lessonae* females result exclusively in *P. esculentus* females, due to the presence of clonal *ridibundus* genomes in the sperm that obviously contain female-determining factors [21, 22, 63].

Two lines of evidence support our finding that *P. esculentus* males from the upper Oder River valley are fertile and maintain the all-male hybrid lineage, by crossing with syntopic *P. ridibundus* females, i.e., they do not originate from primary hybridizations between their parental sexual species, or between sexual males and hybrid females.

First, our extensive sampling did not reveal the presence of *P. lessonae* or even a single hybrid female, necessary for the origin of diploid hybrid males [21]. The second line of evidence comes from independent statistical tests of allelic variation of the nuclear loci. The individually specific MLGs and the presence of both sexes indicate that both investigated taxa, *P. ridibundus* and *P. lessonae*, came from randomly mating sexual populations. *Ridibundus* alleles of *P. esculentus* males were grouped together with alleles of *P. ridibundus* males and females in two different clustering approaches (Fig. 2a, b). Moreover, 27 *ridibundus* genomes from *P. esculentus* males represent 27 MLGs, i.e., again all combinations are unique. It is therefore reasonable to assume that *P. esculentus* received its haploid *ridibundus* genomes from sympatric *P. ridibundus* females. On the other hand, we suggest that the *lessonae* genomes found in *P. esculentus* males from the Oder River are transmitted clonally only by these hybrids over the generations because the *lessonae* genome of *P. esculentus* males differed not only from the genome of sympatric *P. ridibundus* individuals but also from the genome of *P. lessonae* included in this study (Fig. 2a, b). The statistical test for the *P*_sex_ value of these 27 *lessonae* genomes (1.50E^−41^) supports its clonal inheritance.

### All-male hybrids represent a single hemiclone

Hemiclonal hybrids are genetically identical for half of the diploid parental genome [64] which they clonally pass on to the next generation. Analyses of *ridibundus* and *lessonae* allelic richness in all-male *P. esculentus* revealed that 13 loci within the *ridibundus* genome were polymorphic with the observed allelic frequencies (0.036–0.842). Together with the abovementioned observation that single *ridibundus* MLGs were not shared among individuals at all, it is reasonable to assume that these *ridibundus* genomes came from recombinant eggs of sexually reproducing *P. ridibundus*. In the *lessonae* genome, however, we observed only monomorphic loci with allele frequencies of 1.00. Therefore, it is proven that the *lessonae* genome is clonally inherited and represents a single clone (Figs. 2 and 3, Additional file 2: Table S2).

The characteristic of the *lessonae* genome ODERL1 only producing male hybrids is in accordance with the XX/XY type of sex determination hypothesized for the R-E system much earlier by Uzzell et al. [9]. However, our finding that all hybrid males possess an identical *lessonae* (ODERL1) is new finding. Most animal systems with female unisexuality comprise multiple clonal MLGs, as spined loach fishes of the genera *Cobitis* [65] and *Poeciliopsis* [66] and the *Phoxinus eos-neogaeus* complex [67]. Even where hybrids form a monophyletic group, e.g., *Poecilia formosa*, populations show a fairly high level of clonal diversity [68]. In *Pelophylax* populations of the L-E system, where both hybrid males and females coexist, the diversity of transmitted *ridibundus* clones is high [48, 69].

### Origin of the hemiclonal line

Apparently, the existence and maintenance of all-male unisexuality among animals in general, and in *Pelophylax* water frogs in particular, hinge on the formation of a specific genome from a particular sexual species. In European water frogs, the genome of *P. lessonae* represented by the MLG ODERL1 seems to play a key role in the formation of all-male *P. esculentus* lineages. Supporting evidence comes from rare Central- and West-European mixed populations, where all-male *P. esculentus* are triploid and originate from clonal sperm with a diploid *lessonae* genome and haploid *ridibundus* eggs [26].

Despite the evidence for a single origin of the ODERL1 clone and the fact that it has evolved from the *P. lessonae* genepool, this clone does not cluster with other *lessonae-specific* MLGs (Fig. 2a, c) indicating clear differences between the ODERL1 clone and other *P. lessonae* genomes. Due to high distance between the two datasets of *lessonae* alleles, we hypothesize that present *P. lessonae* populations inhabiting areas close to the Oder River valley are not the most recent donors of the *lessonae* genome found in the all-male hybrid lineage of the R-E system populations investigated. It also seems unlikely that the *lessonae* clone recently originated from local *P. lessonae*. Because microsatellite markers have high mutation rates [70], an old hemiclone would comprise several MLGs that would merge into one multilocus lineage [65]. In contrast to this, we found a single *lessonae*-specific MLGs in all hybrids males, indicating a quite recent hybridization event. On the other hand, we cannot exclude a potential selective sweep in the lineage that might be caused by positive natural selection for the ODERL1 hemiclone [65]. Although age estimates of the ODERL1 clone and comparison with more distant *P. lessonae* populations are yet to be obtained, we hypothesize either an older in situ origin or a possible ex situ origin of the ODERL1 clone, i.e., some distance away from the local *P. lessonae* genepool.

## Conclusions

By studying natural populations of *Pelophylax* water frogs along the upper Oder River valley, we discovered a clonal *lessonae* genome represented by a single hemiclone that is present in an all-male *P. esculentus* lineage living in sympatry with *P. ridibundus* males and females. This reproductive mode mirrors the one that previously has been identified in some all-female hybrid animals [5, 6, 71]. This hybridogenetic system in which all-male hybrids coexist with sexual males and females offers intriguing opportunities to compare evolutionary forces and genetic factors forming mating systems that may reverse those operating in all-female systems. These include phenomena like egg-dependent instead of sperm-dependent reproduction or male-male rather than female-female competition over the gamete donors. Additionally, this natural system provides a comparative model to a hemiclonal laboratory system developed in *Drosophila melanogaster* [72] for estimating quantitative genetic parameters in hemiclonal analyses [64]. Elucidating the mechanisms underlying these peculiarities might shed more light on the general processes of evolution of sex or mate-choice theory and contribute to understanding the origin and maintenance of sexual host-parasite dynamics.

## Additional files

Additional file 1: **Table S1.** Numbers of individual genomes included in datasets for a given analysis. (PDF 335 kb)

Additional file 2: **Table S2.** DNA microsatellite data file for the 17 loci used in the study. (PDF 996 kb)

Additional file 3: **Table S3.** Allozyme data file for the six loci used in the study. (PDF 514 kb)

Additional file 4: **Table S4.** Species-specificity of microsatellite alleles used in this study. (PDF 259 kb)

Additional file 5: **Table S5.** Summary statistics of microsatellite alleles found in *P. lessonae*, *P. ridibundus*, and *P. esculentus* from the R-E system. (PDF 317 kb)

Additional file 6: **Figure S1:** Phylogenetic tree of DA distance of 10 microsatellite loci reconstructed in Populations (method UPGMA, 7 000 replicates, shown only bootstraps above 50 %, distance scale). One terminal branch represents one individual: Green color – P. lessonae, yellow color - L genome from P. esculentus, red colour – P. ridibundus, blue color – R genome from P. esculentus, violet color – P. kurtmuelleri. (JPEG 3388 kb)

Additional file 7: **Table S6.** Multilocus Genotypes (MLGs) derived from *P. lessonae*, *P. ridibundus* and *P. esculentus* from the R-E system based on 17 microsatellite loci. (PDF 316 kb)
