# Supplementary material for: All-male hybrids of a tetrapod Pelophylax esculentus share its origin and genetics of maintenance
Source: Biol Sex Differ. 2018 Apr 2;9:13. doi: 10.1186/s13293-018-0172-z (PMC5880063; doi:10.1186/s13293-018-0172-z)
Supplement: Supplementary file 1 — Table S1. Numbers of individual genomes included in datasets for a given analysis. (PDF 335 kb) [file 13293_2018_172_MOESM1_ESM.pdf]

Table S1: Numbers of individual genomes included in data sets for a given analysis.

This Table lists number of loci (No. of loci) and number of diploid genomes from *P. lessonae* (LL), *P. ridibundus* (RR), *P. esculentus* (RL) and *P. kurtmuellleri* (*P. kurt*.) individuals used in different analysis

**Analysis**	**No. of loci**	**All genomes**	**LL**	**RR**	**RL from R-E (L/R)**	**RL from L-E (L/R)**	***P. kurt***
**MLG**	17	204 MLG	108	42	27/27	0/0	0
**PCA**	17	275 MLG	108	42	27/27	71/0	0
**Psex**	10	143 MLG	85	22	21/15	0/0	0
**Structure**	17	205 MLG	108	42	27/27	0/0	1
**Summary statistics**	17	204 MLG	108	42	27/27	0/0	0
**UPGMA**	10	205 MLG	108	42	27/27	0/0	1

LL, *P. lessonae*; RR, *P. ridibundus*; RL, *P. esculentus*; R-E, *P. ridibundus - P. esculentus* population; L-E, *P. lessonae - P. esculentus* population; *P. kurt*, *Pelophylax kurtmuelleri*.
