# Supplementary material for: All-male hybrids of a tetrapod Pelophylax esculentus share its origin and genetics of maintenance
Source: Biol Sex Differ. 2018 Apr 2;9:13. doi: 10.1186/s13293-018-0172-z (PMC5880063; doi:10.1186/s13293-018-0172-z)
Supplement: Supplementary file 2 — Table S2. DNA microsatellite data file for the 17 loci used in the study. (PDF 996 kb) [file 13293_2018_172_MOESM2_ESM.pdf]

Tab S2: DNA microsatellite data file for the 17 loci used in the study.
Description: This Table lists the alleles found in each sampled individual (specified by a respective ID number) on the basis of 17 microsatellite markers.

Sample ID	Population	Taxon	Microsatellite locus					
			RICA1b6		RICA1b5		Ga1a19	
			Allele1	Allele2	Allele1	Allele2	Allele1	Allele2
BI-6-LL	Bravantice	LL	78	78	118	118	195	195
BT-10-LL	Horní Bludovice	LL	80	80	118	118	195	195
BT-4-LL	Horní Bludovice	LL	80	80	118	118	195	195
BT-5-LL	Horní Bludovice	LL	80	80	118	118	195	195
BT-6-LL	Horní Bludovice	LL	78	80	118	118	195	195
BT-7-LL	Horní Bludovice	LL	80	80	118	118	195	195
BT-8-LL	Horní Bludovice	LL	80	80	118	118	195	195
BT-9-LL	Horní Bludovice	LL	80	80	118	118	195	195
CT-10-LL	Český Těšín	LL	80	80	118	118	195	195
CT-11-LL	Český Těšín	LL	80	80	118	118	195	195
CT-1-LL	Český Těšín	LL	80	80	118	118	195	195
CT-2-LL	Český Těšín	LL	80	80	118	118	195	195
CT-3-LL	Český Těšín	LL	80	80	118	118	195	195
CT-4-LL	Český Těšín	LL	80	80	118	118	195	195
CT-5-LL	Český Těšín	LL	78	80	118	118	195	195
CT-6-LL	Český Těšín	LL	78	78	118	118	195	195
CT-7-LL	Český Těšín	LL	78	80	118	118	195	195
CT-8-LL	Český Těšín	LL	78	80	118	118	195	195
DS-1-LL	Důl Staříč	LL	80	80	118	118	195	195
DS-2-LL	Důl Staříč	LL	78	78	118	118	195	195
HB-10-LL	Horní Bludovice	LL	80	83	118	118	195	195
HB-11-LL	Horní Bludovice	LL	80	80	118	118	195	195
HB-12-LL	Horní Bludovice	LL	80	80	118	118	195	195
HB-13-LL	Horní Bludovice	LL	78	80	0	0	0	0
HB-14-LL	Horní Bludovice	LL	80	80	0	0	195	195
HB-15-LL	Horní Bludovice	LL	80	83	118	134	195	195
HB-1-LL	Horní Bludovice	LL	80	80	118	118	195	195
HB-2-LL	Horní Bludovice	LL	78	80	118	118	195	195
HB-3-LL	Horní Bludovice	LL	80	80	118	118	195	195
HB-4-LL	Horní Bludovice	LL	78	78	118	118	195	195
HB-5-LL	Horní Bludovice	LL	78	78	118	118	195	195
HB-6-LL	Horní Bludovice	LL	78	80	118	118	195	195
HB-7-LL	Horní Bludovice	LL	78	78	118	118	195	195
HB-8-LL	Horní Bludovice	LL	78	80	118	118	195	195
HD-12-LL	Horní Domaslavice	LL	78	80	118	118	195	195
HD-13-LL	Horní Domaslavice	LL	78	80	118	118	195	195
HD-14-LL	Horní Domaslavice	LL	78	80	118	118	195	195
HD-15-LL	Horní Domaslavice	LL	80	83	118	118	195	195
HD-4-LL	Horní Domaslavice	LL	78	83	118	118	195	195
HD-6-LL	Horní Domaslavice	LL	78	80	118	118	195	195
HD-9-LL	Horní Domaslavice	LL	80	80	118	118	195	195
Ka-13-LL	Karviná-Doly	LL	80	80	118	118	195	195
Ka-9-LL	Karviná-Doly	LL	80	80	118	118	195	195

LO-9-LL	Louky	LL	80	80	118	118	195	195
PR-11-LL	Prstná	LL	78	80	118	118	195	195
PR-2-LL	Prstná	LL	78	78	118	134	195	201
PR-3-LL	Prstná	LL	80	80	118	118	195	195
PR-4-LL	Prstná	LL	78	80	118	118	195	195
PR-5-LL	Prstná	LL	78	80	118	118	195	195
PR-6-LL	Prstná	LL	80	80	118	118	195	195
PR-7-LL	Prstná	LL	78	80	118	118	195	195
PR-8-LL	Prstná	LL	80	80	118	118	195	195
TR-10-LL	Trnávka	LL	78	78	118	118	195	195
TR-11-LL	Trnávka	LL	80	83	118	118	195	195
TR-12-LL	Trnávka	LL	78	80	118	118	195	195
TR-13-LL	Trnávka	LL	78	80	118	118	195	195
TR-14-LL	Trnávka	LL	80	83	118	118	195	195
TR-15-LL	Trnávka	LL	78	80	118	118	195	195
TR-3-LL	Trnávka	LL	78	80	118	118	195	195
TR-4-LL	Trnávka	LL	78	78	118	118	195	195
TR-5-LL	Trnávka	LL	78	80	118	118	195	195
TR-8-LL	Trnávka	LL	78	80	118	118	195	195
TR-9-LL	Trnávka	LL	78	80	118	118	195	195
DO-91-LL	Dobrá	LL	78	80	118	118	195	195
DO-92-LL	Dobrá	LL	78	80	118	118	195	195
DO-93-LL	Dobrá	LL	80	80	118	118	195	195
DO-94-LL	Dobrá	LL	78	80	118	118	195	195
DO-95-LL	Dobrá	LL	78	80	118	118	195	195
DO-01-LL	Dobrá	LL	80	80	118	118	195	195
DO-02-LL	Dobrá	LL	78	80	118	118	195	195
DO-03-LL	Dobrá	LL	78	80	118	118	195	195
DO-24-LL	Dobrá	LL	80	80	118	118	195	195
DO-25-LL	Dobrá	LL	80	80	118	118	195	195
DO-26-LL	Dobrá	LL	78	80	118	118	195	195
DO-27-LL	Dobrá	LL	78	80	118	118	195	195
DO-28-LL	Dobrá	LL	80	80	118	118	195	195
DO-29-LL	Dobrá	LL	78	83	118	118	195	195
DO-30-LL	Dobrá	LL	80	80	118	118	195	195
DO-31-LL	Dobrá	LL	80	80	118	118	195	195
DO-32-LL	Dobrá	LL	78	80	118	118	195	195
DO-33-LL	Dobrá	LL	78	78	113	118	195	195
DO-34-LL	Dobrá	LL	80	83	118	118	195	195
DO-35-LL	Dobrá	LL	78	80	118	118	195	195
DO-36-LL	Dobrá	LL	78	83	113	118	195	195
DO-37-LL	Dobrá	LL	80	83	118	118	195	195
DO-38-LL	Dobrá	LL	78	78	118	118	195	195
DO-39-LL	Dobrá	LL	78	80	118	118	195	195
DO-40-LL	Dobrá	LL	80	83	118	118	195	195
DO-41-LL	Dobrá	LL	78	80	118	118	195	195
DO-42-LL	Dobrá	LL	80	83	118	118	195	195
DO-44-LL	Dobrá	LL	80	80	118	118	195	195
DO-46-LL	Dobrá	LL	78	80	118	118	195	195
DO-47-LL	Dobrá	LL	80	80	118	118	195	195

DO-48-LL	Dobrá	LL	80	80	118	118	195	195
TR-26-LL	Trnávka	LL	78	83	118	118	195	195
TR-29-LL	Trnávka	LL	80	80	118	118	195	195
TR-30-LL	Trnávka	LL	80	80	118	118	195	195
TR-40-LL	Trnávka	LL	78	80	118	118	195	195
TR-49-LL	Trnávka	LL	80	80	118	118	195	195
TR-53-LL	Trnávka	LL	78	80	118	118	195	195
TR-66-LL	Trnávka	LL	78	80	118	118	195	195
TR-67-LL	Trnávka	LL	78	80	118	118	195	195
TR-68-LL	Trnávka	LL	78	80	118	118	195	195
TR-69-LL	Trnávka	LL	78	80	118	118	195	195
BD-95-LL	Břidličná	LL	80	80	118	118	195	195
BD-01-LL	Břidličná	LL	78	78	118	118	195	195
BD-08-LL	Břidličná	LL	78	80	118	118	195	195
BD-16-LL	Břidličná	LL	78	80	118	118	195	195
BI-10-RL	Bravantice	RL	80	NA	118	NA	195	NA
BI-12-RL	Bravantice	RL	80	NA	118	NA	195	NA
BI-2-RL	Bravantice	RL	80	NA	118	NA	195	NA
BI-3-RL	Bravantice	RL	80	NA	118	NA	195	NA
BI-4-RL	Bravantice	RL	80	NA	118	NA	195	NA
BI-5-RL	Bravantice	RL	78	NA	118	NA	195	NA
BI-9-RL	Bravantice	RL	78	NA	118	NA	195	NA
BT-1-RL	Horní Bludovice	RL	78	NA	118	NA	195	NA
BT-2-RL	Horní Bludovice	RL	80	NA	118	NA	195	NA
BT-3-RL	Horní Bludovice	RL	80	NA	118	NA	195	NA
CT-12-RL	Český Těšín	RL	80	NA	118	NA	195	NA
CT-13-RL	Český Těšín	RL	78	NA	118	NA	195	NA
CT-14-RL	Český Těšín	RL	78	NA	118	NA	195	NA
CT-15-RL	Český Těšín	RL	80	NA	118	NA	195	NA
CT-9-RL	Český Těšín	RL	78	NA	118	NA	195	NA
DS-10-RL	Důl Staříč	RL	80	NA	118	NA	195	NA
DS-11-RL	Důl Staříč	RL	80	NA	118	NA	195	NA
DS-12-RL	Důl Staříč	RL	80	NA	118	NA	195	NA
DS-13-RL	Důl Staříč	RL	80	NA	118	NA	195	NA
DS-14-RL	Důl Staříč	RL	78	NA	118	NA	195	NA
DS-15-RL	Důl Staříč	RL	80	NA	0	NA	195	NA
DS-3-RL	Důl Staříč	RL	83	NA	0	NA	195	NA
DS-4-RL	Důl Staříč	RL	80	NA	118	NA	195	NA
DS-5-RL	Důl Staříč	RL	80	NA	118	NA	0	NA
DS-6-RL	Důl Staříč	RL	80	NA	118	NA	195	NA
DS-7-RL	Důl Staříč	RL	78	NA	118	NA	195	NA
DS-8-RL	Důl Staříč	RL	83	NA	118	NA	195	NA
DS-9-RL	Důl Staříč	RL	80	NA	118	NA	195	NA
HB-9-RL	Horní Bludovice	RL	80	NA	118	NA	0	NA
HD-10-RL	Horní Domaslavice	RL	83	NA	118	NA	195	NA
HD-11-RL	Horní Domaslavice	RL	80	NA	118	NA	195	NA
HD-1-RL	Horní Domaslavice	RL	80	NA	118	NA	195	NA
HD-2-RL	Horní Domaslavice	RL	80	NA	118	NA	195	NA
HD-3-RL	Horní Domaslavice	RL	78	NA	118	NA	195	NA
HD-5-RL	Horní Domaslavice	RL	80	NA	118	NA	195	NA

HD-7-RL	Horní Domaslavice	RL	80	NA	118	NA	195	NA
HD-8-RL	Horní Domaslavice	RL	83	NA	118	NA	195	NA
Ka-10-RL	Karviná-Doly	RL	80	NA	118	NA	195	NA
Ka-11-RL	Karviná-Doly	RL	78	NA	118	NA	195	NA
Ka-12-RL	Karviná-Doly	RL	80	NA	118	NA	195	NA
Ka-14-RL	Karviná-Doly	RL	78	NA	118	NA	195	NA
Ka-15-RL	Karviná-Doly	RL	80	NA	118	NA	195	NA
Ka-1-RL	Karviná-Doly	RL	80	NA	118	NA	195	NA
Ka-2-RL	Karviná-Doly	RL	80	NA	118	NA	195	NA
Ka-3-RL	Karviná-Doly	RL	80	NA	118	NA	195	NA
Ka-4-RL	Karviná-Doly	RL	80	NA	118	NA	195	NA
Ka-5-RL	Karviná-Doly	RL	80	NA	118	NA	195	NA
Ka-6-RL	Karviná-Doly	RL	78	NA	118	NA	195	NA
Ka-7-RL	Karviná-Doly	RL	80	NA	118	NA	195	NA
Ka-8-RL	Karviná-Doly	RL	78	NA	118	NA	195	NA
LO-10-RL	Louky	RL	80	NA	118	NA	195	NA
LO-11-RL	Louky	RL	80	NA	118	NA	195	NA
LO-12-RL	Louky	RL	80	NA	118	NA	195	NA
LO-13-RL	Louky	RL	80	NA	118	NA	195	NA
LO-14-RL	Louky	RL	80	NA	118	NA	195	NA
LO-15-RL	Louky	RL	78	NA	118	NA	195	NA
LO-1-RL	Louky	RL	80	NA	118	NA	195	NA
LO-2-RL	Louky	RL	80	NA	118	NA	195	NA
LO-3-RL	Louky	RL	80	NA	118	NA	195	NA
LO-4-RL	Louky	RL	80	NA	118	NA	195	NA
LO-5-RL	Louky	RL	78	NA	118	NA	195	NA
LO-6-RL	Louky	RL	80	NA	118	NA	195	NA
LO-7-RL	Louky	RL	80	NA	118	NA	195	NA
LO-8-RL	Louky	RL	80	NA	118	NA	195	NA
PR-10-RL	Prstná	RL	80	NA	118	NA	195	NA
PR-1-RL	Prstná	RL	80	NA	118	NA	195	NA
PR-9-RL	Prstná	RL	80	NA	118	NA	195	NA
TR-1-RL	Trnávka	RL	80	NA	118	NA	195	NA
TR-2-RL	Trnávka	RL	80	NA	118	NA	195	NA
TR-6-RL	Trnávka	RL	80	NA	118	NA	195	NA
TR-7-RL	Trnávka	RL	80	NA	118	NA	195	NA
PO-75-RL	Albrechtičky	RL	78	92	118	134	195	201
PO-82-RL	Albrechtičky	RL	78	94	118	134	195	223
PO-84-RL	Albrechtičky	RL	78	92	118	134	195	205
BI-7-RL	Bílovec	RL	78	92	118	134	195	205
BI-8-RL	Bílovec	RL	78	92	118	134	195	201
Da-10-RL	Darkovice	RL	78	92	118	134	195	201
Da-11-RL	Darkovice	RL	78	85	118	134	195	205
Da-12-RL	Darkovice	RL	78	92	118	134	195	247
Da-9-RL	Darkovice	RL	78	92	118	134	195	201
DB-10-RL	Dolní Benešov	RL	78	85	118	134	195	205
DB-1-RL	Dolní Benešov	RL	78	92	118	134	195	201
DB-4-RL	Dolní Benešov	RL	78	92	118	134	195	243
DB-5-RL	Dolní Benešov	RL	78	92	118	134	195	223
OS-10-RL	Ostrava	RL	78	92	118	134	195	205

OS-11-RL	Ostrava	RL	78	92	118	134	195	201
OS-12-RL	Ostrava	RL	78	85	118	134	195	201
OS-14-RL	Ostrava	RL	78	92	118	136	195	205
OS-6-RL	Ostrava	RL	78	83	118	134	195	201
PO-10-RL	Albrechtičky	RL	78	94	118	134	195	201
PO-14-RL	Albrechtičky	RL	78	92	118	134	195	243
PO-15-RL	Albrechtičky	RL	78	83	0	0	195	0
PO-1-RL	Albrechtičky	RL	78	85	118	134	195	209
PO-2-RL	Albrechtičky	RL	78	92	118	134	195	201
PO-3-RL	Albrechtičky	RL	78	92	118	132	195	201
PO-7-RL	Albrechtičky	RL	78	92	118	0	0	0
PO-8-RL	Albrechtičky	RL	78	94	118	134	195	201
PO-9-RL	Albrechtičky	RL	78	85	118	134	195	201
BI-11-RR	Bílovec	RR	92	94	134	134	201	259
Da-1-RR	Darkovice	RR	85	92	134	134	201	247
Da-2-RR	Darkovice	RR	92	98	134	134	205	205
Da-3-RR	Darkovice	RR	92	92	134	134	201	247
Da-4-RR	Darkovice	RR	85	92	134	134	201	247
Da-5-RR	Darkovice	RR	85	92	134	134	201	247
Da-6-RR	Darkovice	RR	92	92	134	134	201	247
Da-7-RR	Darkovice	RR	92	92	134	134	201	247
Da-8-RR	Darkovice	RR	85	92	134	134	201	201
DB-2-RR	Dolní Benešov	RR	92	92	134	138	201	243
DB-3-RR	Dolní Benešov	RR	85	92	134	134	205	205
DB-6-RR	Dolní Benešov	RR	92	92	134	134	243	243
DB-7-RR	Dolní Benešov	RR	0	0	134	134	0	0
DB-8-RR	Dolní Benešov	RR	74	92	134	134	201	243
DB-9-RR	Dolní Benešov	RR	85	85	134	134	201	243
OS-13-RR	Ostrava	RR	83	85	134	136	201	217
OS-1-RR	Ostrava	RR	83	92	134	134	205	209
OS-2-RR	Ostrava	RR	85	92	134	134	201	205
OS-3-RR	Ostrava	RR	78	92	134	134	201	253
OS-4-RR	Ostrava	RR	92	92	134	134	201	205
OS-5-RR	Ostrava	RR	92	98	134	134	201	255
OS-7-RR	Ostrava	RR	92	92	134	134	201	205
OS-8-RR	Ostrava	RR	78	92	134	134	205	253
OS-9-RR	Ostrava	RR	85	92	134	136	201	205
PO-11-RR	Albrechtičky	RR	94	94	134	134	201	243
PO-12-RR	Albrechtičky	RR	92	94	134	134	239	247
PO-13-RR	Albrechtičky	RR	92	98	134	134	201	201
PO-4-RR	Albrechtičky	RR	83	92	134	134	201	217
PO-5-RR	Albrechtičky	RR	92	92	134	134	205	205
TR-65-RR	Albrechtičky	RR	92	94	134	134	201	243
PO-70-RR	Albrechtičky	RR	92	92	134	136	201	201
PO-71-RR	Albrechtičky	RR	92	92	134	134	205	217
PO-72-RR	Albrechtičky	RR	85	94	132	134	223	223
PO-73-RR	Albrechtičky	RR	85	92	134	134	205	217
PO-74-RR	Albrechtičky	RR	94	94	134	134	205	247
PO-76-RR	Albrechtičky	RR	85	98	134	134	243	243
PO-77-RR	Albrechtičky	RR	85	85	134	134	205	223

PO-78-RR	Albrechtičky	RR	94	94	134	134	205	205
PO-79-RR	Albrechtičky	RR	92	92	134	134	201	205
PO-80-RR	Albrechtičky	RR	92	92	134	134	201	201
PO-81-RR	Albrechtičky	RR	85	92	134	134	217	249
PO-83-RR	Albrechtičky	RR	83	92	134	134	201	205
GR07-R1	Greece	RR	87	104	132	132	197	205

Notes: RR, *P. ridibundus;* RL, *P. esculentus;* LL, *P. lessonae* ; NA, not analysed.

RICA5		Res16		Res20		RICA2a3		Re2Caga		Res22	
Allele1	Allele2	Allele1	Allele2	Allele1	Allele2	Allele1	Allele2	Allele1	Allele2	Allele1	Allele2
260	260	121	121	124	0	145	145	0	0	0	0
256	256	121	121	120	126	145	147	0	0	0	0
256	260	121	121	120	126	145	147	0	0	0	0
256	256	121	121	110	116	145	147	0	0	0	0
256	264	121	121	128	0	145	150	0	0	0	0
256	256	121	121	126	0	145	150	0	0	0	0
252	260	121	121	116	126	145	147	0	0	0	0
256	260	121	121	116	126	145	147	0	0	0	0
256	262	121	121	120	0	138	147	0	0	110	0
256	256	121	121	120	0	145	152	0	0	0	0
256	256	121	121	116	0	147	150	0	0	0	0
256	256	121	121	108	116	145	147	0	0	0	0
256	256	121	121	120	142	145	147	0	0	0	0
0	0	121	121	110	120	145	150	0	0	0	0
256	256	121	121	110	120	147	150	0	0	0	0
256	262	121	121	108	110	147	154	0	0	0	0
260	260	121	121	108	110	112	130	0	0	0	0
256	256	121	121	110	0	145	145	0	0	0	0
260	262	121	121	120	0	112	138	0	0	0	0
0	0	121	121	116	120	112	145	0	0	0	0
256	256	121	121	120	126	145	152	0	0	110	0
260	260	121	121	116	126	145	147	0	0	0	0
0	0	0	0	0	0	0	0	0	0	0	0
0	0	121	121	120	0	145	145	0	0	83	110
256	256	121	121	110	120	145	147	0	0	0	0
0	0	121	121	120	0	140	145	0	0	110	0
252	256	121	121	120	0	140	145	0	0	0	0
256	256	121	121	116	120	145	145	0	0	0	0
256	256	121	121	110	116	145	145	0	0	0	0
256	256	121	121	120	0	145	145	0	0	0	0
0	0	121	121	116	120	147	162	0	0	0	0
252	256	121	121	124	0	145	147	0	0	0	0
252	260	121	121	110	0	145	147	0	0	0	0
256	258	121	121	108	110	145	145	0	0	0	0
256	256	121	121	120	126	140	143	0	0	0	0
256	256	121	121	112	120	140	145	0	0	0	0
256	256	121	121	104	110	112	145	0	0	0	0
256	260	121	121	120	124	145	152	0	0	0	0
256	256	121	121	110	120	145	152	0	0	110	0
256	256	121	121	110	0	140	145	0	0	0	0
256	260	121	121	120	120	140	152	0	0	0	0
256	256	121	121	110	120	147	156	0	0	0	0
256	260	121	121	108	110	136	145	0	0	0	0

256	260	121	121	108	110	140	145	0	0	110	0
256	260	121	121	110	120	145	152	0	0	110	0
252	256	121	121	116	126	140	145	0	0	110	0
256	256	121	121	110	0	123	147	0	0	110	0
256	256	121	121	120	122	150	164	0	0	0	0
256	260	121	121	110	116	147	152	0	0	0	0
256	256	121	121	110	120	140	145	0	0	0	0
256	260	121	121	110	131	147	147	0	0	0	0
256	256	121	121	110	116	147	150	0	0	0	0
260	260	121	121	142	144	145	147	0	0	0	0
256	256	121	121	110	124	145	145	0	0	0	0
256	256	121	121	110	0	138	145	0	0	0	0
260	260	121	121	110	120	145	152	0	0	0	0
256	260	121	121	110	120	112	140	235	0	0	0
252	260	121	121	124	142	154	154	0	0	0	0
260	260	121	121	110	146	152	154	231	0	110	0
256	260	121	121	146	146	145	152	0	0	0	0
260	260	121	121	120	120	140	147	0	0	110	0
260	260	121	121	120	120	145	147	0	0	110	0
256	260	121	121	142	142	150	152	0	0	0	0
247	256	121	121	110	0	136	145	0	0	0	0
256	260	152	0	116	0	145	162	0	0	0	0
256	256	121	121	110	0	138	150	0	0	0	0
256	256	121	121	120	120	136	140	0	0	0	0
256	260	121	121	120	120	145	162	0	0	0	0
256	256	121	121	102	110	145	145	0	0	0	0
256	256	121	121	120	120	145	145	0	0	0	0
256	260	121	121	102	110	145	145	0	0	0	0
256	260	121	121	128	128	140	145	0	0	0	0
256	260	121	121	110	124	145	145	0	0	0	0
260	260	121	121	110	114	145	147	0	0	0	0
256	256	121	121	116	128	136	145	0	0	0	0
260	260	121	121	122	128	145	145	0	0	0	0
256	260	0	0	110	124	140	140	0	0	0	0
256	260	121	121	110	128	145	152	0	0	0	0
256	260	121	121	110	124	140	162	0	0	0	0
256	264	121	121	110	0	136	147	0	0	0	0
256	264	121	121	110	0	140	143	0	0	0	0
256	256	121	121	128	128	145	147	0	0	0	0
256	256	121	121	110	110	147	154	0	0	0	0
256	260	121	121	110	124	140	145	0	0	0	0
256	260	121	121	116	0	145	147	0	0	0	0
256	256	121	121	110	110	140	145	0	0	0	0
260	260	121	121	116	116	145	147	0	0	0	0
256	256	0	0	116	128	145	147	0	0	0	0
260	260	121	121	110	114	150	152	0	0	0	0
256	260	121	121	120	120	145	150	0	0	0	0
256	260	121	121	110	124	140	147	0	0	0	0
247	256	121	121	110	116	147	162	0	0	0	0
256	264	121	121	110	110	136	145	0	0	0	0

256	260	121	121	120	124	136	136	0	0	0	0
256	260	121	121	112	120	145	150	0	0	0	0
256	260	121	121	110	110	147	150	0	0	0	0
260	260	121	121	110	110	145	150	0	0	0	0
256	260	121	121	142	144	145	152	0	0	0	0
256	256	121	121	0	0	145	152	0	0	0	0
256	256	121	121	0	0	145	145	0	0	0	0
256	260	121	121	112	114	136	147	0	0	0	0
256	260	121	121	110	110	112	145	0	0	0	0
260	260	121	121	110	110	145	145	0	0	0	0
256	260	121	121	120	120	145	147	0	0	0	0
260	260	121	133	118	118	112	145	0	0	0	0
252	256	121	121	118	124	143	145	0	0	98	110
260	260	121	121	110	124	145	145	0	0	0	0
258	260	121	121	122	124	145	145	0	0	0	0
256	NA	121	NA	120	NA	143	NA	0	NA	0	NA
256	NA	121	NA	120	NA	145	NA	0	NA	0	NA
256	NA	121	NA	110	NA	112	NA	0	NA	0	NA
0	NA	121	NA	110	NA	120	NA	0	NA	0	NA
260	NA	121	NA	110	NA	145	NA	0	NA	0	NA
256	NA	0	NA	110	NA	145	NA	0	NA	0	NA
0	NA	121	NA	110	NA	112	NA	0	NA	0	NA
256	NA	121	NA	120	NA	154	NA	0	NA	0	NA
260	NA	121	NA	120	NA	145	NA	0	NA	0	NA
256	NA	121	NA	120	NA	145	NA	0	NA	0	NA
0	NA	0	NA	116	NA	145	NA	0	NA	0	NA
256	NA	121	NA	0	NA	138	NA	0	NA	0	NA
262	NA	0	NA	122	NA	145	NA	0	NA	0	NA
256	NA	121	NA	114	NA	140	NA	0	NA	0	NA
256	NA	121	NA	0	NA	145	NA	0	NA	0	NA
256	NA	121	NA	120	NA	145	NA	0	NA	0	NA
256	NA	121	NA	120	NA	154	NA	0	NA	0	NA
264	NA	0	NA	146	NA	147	NA	0	NA	0	NA
0	NA	0	NA	0	NA	0	NA	0	NA	0	NA
260	NA	121	NA	110	NA	145	NA	0	NA	0	NA
256	NA	121	NA	120	NA	145	NA	0	NA	0	NA
0	NA	121	NA	0	NA	147	NA	0	NA	0	NA
260	NA	121	NA	120	NA	145	NA	0	NA	0	NA
0	NA	0	NA	0	NA	0	NA	0	NA	0	NA
254	NA	121	NA	108	NA	145	NA	0	NA	0	NA
260	NA	121	NA	142	NA	147	NA	0	NA	0	NA
260	NA	121	NA	120	NA	147	NA	0	NA	0	NA
256	NA	121	NA	144	NA	147	NA	0	NA	0	NA
0	NA	121	NA	120	NA	145	NA	0	NA	0	NA
256	NA	121	NA	110	NA	152	NA	0	NA	0	NA
256	NA	121	NA	126	NA	152	NA	0	NA	0	NA
260	NA	121	NA	110	NA	162	NA	0	NA	0	NA
256	NA	121	NA	110	NA	150	NA	0	NA	0	NA
256	NA	121	NA	120	NA	143	NA	0	NA	0	NA
256	NA	121	NA	126	NA	145	NA	0	NA	0	NA

256	NA	121	NA	116	NA	145	NA	0	NA	0	NA
256	NA	121	NA	126	NA	145	NA	0	NA	0	NA
256	NA	121	NA	120	NA	136	NA	0	NA	0	NA
256	NA	121	NA	108	NA	145	NA	0	NA	0	NA
256	NA	121	NA	110	NA	147	NA	0	NA	0	NA
256	NA	121	NA	120	NA	156	NA	0	NA	0	NA
256	NA	121	NA	124	NA	147	NA	0	NA	0	NA
256	NA	121	NA	120	NA	150	NA	0	NA	0	NA
256	NA	121	NA	120	NA	147	NA	0	NA	0	NA
260	NA	121	NA	110	NA	150	NA	0	NA	0	NA
256	NA	121	NA	110	NA	145	NA	0	NA	0	NA
256	NA	121	NA	120	NA	140	NA	0	NA	0	NA
256	NA	121	NA	120	NA	134	NA	0	NA	0	NA
256	NA	121	NA	116	NA	150	NA	0	NA	0	NA
256	NA	121	NA	120	NA	154	NA	0	NA	0	NA
260	NA	121	NA	131	NA	147	NA	0	NA	0	NA
256	NA	121	NA	112	NA	150	NA	0	NA	0	NA
256	NA	121	NA	120	NA	145	NA	0	NA	0	NA
256	NA	121	NA	120	NA	145	NA	0	NA	0	NA
256	NA	121	NA	108	NA	145	NA	0	NA	0	NA
256	NA	121	NA	110	NA	138	NA	0	NA	0	NA
256	NA	121	NA	128	NA	145	NA	0	NA	0	NA
256	NA	121	NA	120	NA	147	NA	0	NA	0	NA
260	NA	121	NA	110	NA	145	NA	0	NA	0	NA
256	NA	121	NA	120	NA	150	NA	0	NA	0	NA
256	NA	121	NA	110	NA	145	NA	0	NA	0	NA
260	NA	121	NA	110	NA	145	NA	0	NA	0	NA
260	NA	121	NA	110	NA	147	NA	0	NA	0	NA
256	NA	121	NA	126	NA	150	NA	0	NA	0	NA
256	NA	121	NA	120	NA	140	NA	0	NA	0	NA
0	NA	121	NA	120	NA	147	NA	0	NA	0	NA
260	NA	121	NA	118	NA	147	NA	0	NA	0	NA
260	NA	121	NA	110	NA	147	NA	0	NA	0	NA
256	NA	121	NA	110	NA	147	NA	0	NA	0	NA
256	NA	121	NA	110	NA	147	NA	0	NA	0	NA
256	NA	121	NA	110	NA	147	NA	0	NA	0	NA
260	0	121	127	120	0	145	0	0	196	0	83
260	0	121	0	120	0	145	106	0	169	0	110
260	0	121	0	120	0	145	110	0	204	0	98
260	256	121	127	120	0	145	0	0	0	0	110
0	0	121	0	120	0	145	106	0	0	0	110
260	232	121	127	120	0	145	0	0	212	0	83
260	234	121	127	120	0	145	106	0	208	0	133
260	232	121	115	120	0	145	106	0	231	0	83
260	0	121	115	120	0	145	0	0	212	0	83
260	0	121	127	120	0	145	106	0	208	0	110
260	0	121	127	120	0	145	110	0	235	0	110
260	0	121	0	120	0	145	0	0	169	0	110
260	0	121	0	120	0	145	0	0	169	0	110
260	236	121	127	120	0	145	110	0	212	0	110

260	0	121	0	120	0	145	106	0	196	0	110
260	0	121	0	120	0	145	106	0	196	0	110
260	0	121	127	120	0	145	110	0	169	0	104
260	0	121	115	120	0	145	106	0	200	0	110
260	0	121	0	120	0	145	110	0	169	0	110
260	0	121	127	120	0	145	106	0	0	0	104
0	0	121	0	120	0	145	0	0	176	0	110
260	0	121	0	120	0	145	106	0	169	0	110
260	0	121	129	120	0	145	110	0	0	0	110
0	0	121	0	120	0	145	110	0	0	0	110
0	0	121	127	120	0	145	106	0	169	0	110
260	0	121	127	120	0	145	106	0	169	0	110
260	0	121	127	120	0	145	106	0	169	0	110
234	0	127	0	0	0	106	106	220	235	110	110
232	0	119	123	0	0	106	106	212	231	87	129
232	250	127	0	0	0	106	110	208	235	83	110
232	0	117	123	0	0	106	106	212	231	87	110
232	260	117	123	0	0	106	106	192	212	87	129
232	0	119	123	0	0	106	106	192	212	83	129
232	0	119	0	0	0	106	106	169	231	83	129
232	0	119	0	0	0	106	106	169	231	83	129
0	0	127	0	0	0	106	110	169	235	110	110
232	248	121	0	0	0	106	106	169	196	110	110
232	248	127	0	0	0	0	0	169	169	110	110
232	260	127	0	0	0	106	106	169	227	110	110
0	0	0	0	0	0	0	0	0	0	110	110
232	0	115	0	0	0	106	110	169	220	110	110
232	250	127	0	0	0	106	106	169	231	110	110
234	236	115	127	0	0	106	106	204	208	110	129
232	232	127	0	0	0	106	106	169	200	83	83
234	234	127	0	0	0	110	110	200	208	110	129
232	232	127	0	0	0	106	106	169	200	110	116
234	234	115	0	0	0	106	106	212	220	110	110
232	232	127	0	0	0	106	106	220	235	106	110
232	250	127	0	0	0	106	110	200	220	83	110
232	232	115	117	0	0	106	106	231	255	110	110
232	234	127	0	0	0	106	106	212	235	83	110
232	232	121	0	0	0	106	110	200	231	110	110
232	232	127	127	0	0	106	110	0	0	106	110
0	0	127	127	0	0	106	106	169	220	110	127
0	0	127	127	0	0	106	106	169	192	104	110
232	232	121	0	0	0	110	110	169	200	83	108
232	232	121	0	0	0	106	110	169	169	110	110
0	0	121	0	0	0	106	110	169	235	110	116
0	0	127	127	0	0	106	106	169	169	110	110
0	0	127	127	0	0	106	106	208	220	98	108
0	0	121	0	0	0	106	106	169	169	114	116
0	0	121	0	0	0	106	106	212	231	83	106
0	0	127	127	0	0	106	110	169	169	104	110
0	0	121	0	0	0	106	106	169	231	110	124

0	0	119	119	0	0	110	110	169	192	110	114
232	232	121	0	0	0	106	106	192	200	110	116
0	0	127	127	0	0	106	106	169	231	110	110
0	0	127	127	0	0	110	110	169	169	110	110
0	0	115	0	0	0	106	106	169	192	110	116
246	258	115	115	0	0	110	110	200	208	81	91

Ga1a23		Rrid169A		Rrid013A		Rrid059A		Re1Caga10		RICA1a27	
Allele1	Allele2	Allele1	Allele2	Allele1	Allele2	Allele1	Allele2	Allele1	Allele2	Allele1	Allele2
113	119	0	0	0	0	0	0	97	97	95	111
119	127	0	0	296	296	278	278	97	97	95	111
119	121	0	0	296	296	278	278	97	97	111	125
119	119	0	0	296	296	278	278	97	97	111	111
119	121	0	0	296	296	278	278	97	97	111	119
0	0	0	0	0	0	0	0	0	0	0	0
119	121	0	0	296	296	278	278	97	97	95	111
121	121	0	0	296	296	278	278	97	97	95	111
119	119	0	0	296	296	278	278	97	97	111	119
117	121	0	0	293	296	278	278	97	97	111	111
113	131	0	0	0	0	0	0	97	97	111	111
131	131	0	0	296	296	278	278	97	97	111	111
123	131	0	0	293	299	278	278	97	97	111	119
113	121	0	0	0	0	278	278	97	97	111	119
117	119	0	0	296	296	278	278	97	97	111	113
123	123	0	0	296	296	278	278	97	97	111	119
117	119	191	0	296	296	278	278	97	97	111	113
119	119	0	0	296	299	278	278	97	97	111	111
119	123	0	0	296	296	278	278	97	97	95	119
119	119	0	0	0	0	0	0	97	97	95	95
121	125	0	0	0	0	0	0	97	97	95	111
119	131	0	0	0	0	0	0	97	97	111	111
119	121	0	0	0	0	0	0	97	97	95	125
119	129	0	0	0	0	0	0	97	97	111	111
119	121	0	0	0	0	0	0	97	97	95	119
125	131	0	0	0	0	0	0	97	97	119	119
121	129	0	0	296	296	278	278	97	97	111	121
119	119	0	0	0	0	0	0	97	97	111	121
125	131	0	0	296	296	278	278	97	97	111	111
119	121	0	0	0	0	0	0	97	97	111	111
119	121	0	0	0	0	0	0	97	97	111	119
119	119	0	0	296	296	0	0	97	97	95	121
119	119	0	0	296	296	278	278	97	97	125	125
119	121	0	0	296	296	0	0	97	97	95	119
119	121	0	0	296	296	278	278	97	97	95	95
119	119	0	0	293	296	278	278	97	97	111	121
0	0	0	0	0	0	0	0	0	0	0	0
119	119	0	0	291	296	278	278	97	97	111	121
119	131	187	0	296	296	278	278	97	97	119	125
0	0	0	0	0	0	0	0	0	0	0	0
113	121	0	0	296	296	278	278	97	97	95	111
0	0	0	0	0	0	0	0	0	0	0	0
119	131	0	0	296	299	278	278	97	97	95	119

119	125	191	234	296	296	278	278	97	97	95	111
98	131	227	0	296	296	278	278	97	97	95	95
98	115	187	0	296	296	278	278	97	106	111	121
98	125	187	227	291	296	278	278	97	116	95	115
119	133	187	0	296	296	278	278	97	97	119	121
119	139	0	0	296	296	278	278	97	97	117	119
125	125	0	0	296	299	278	278	97	97	111	119
115	131	0	0	296	296	278	278	97	97	111	119
0	0	0	0	0	0	0	0	0	0	0	0
115	127	0	0	296	296	278	278	97	97	95	111
123	135	0	0	296	296	278	278	97	97	95	95
123	127	0	0	296	296	278	278	97	97	95	121
119	123	0	0	296	296	278	278	97	97	119	121
131	141	0	0	296	296	278	278	97	116	95	115
0	0	0	0	0	0	0	0	97	97	95	121
115	119	191	227	296	296	278	278	97	106	95	119
123	123	0	0	296	296	278	278	97	97	95	119
127	131	0	0	296	296	278	278	97	97	95	95
127	131	191	0	296	296	278	278	97	97	95	95
127	127	0	0	296	296	278	278	97	97	113	119
121	131	0	0	296	296	278	278	97	97	95	111
119	121	0	0	296	296	278	278	97	97	111	115
123	127	0	0	293	296	278	278	97	97	111	111
121	131	0	0	296	296	278	278	97	97	111	119
127	131	0	0	296	296	278	278	97	97	111	121
121	121	0	0	296	296	278	278	97	97	111	121
119	121	0	0	296	296	278	278	97	97	111	119
121	125	0	0	296	296	278	278	97	97	111	119
119	123	0	0	296	296	278	278	97	97	95	119
125	131	0	0	296	299	278	278	97	97	119	127
131	131	0	0	296	296	278	278	97	97	119	121
119	125	0	0	296	296	278	278	97	97	111	119
119	123	0	0	296	299	278	278	97	97	111	119
131	131	0	0	296	299	278	278	97	97	95	127
119	125	0	0	296	296	278	278	97	97	111	111
119	131	0	0	296	296	278	278	97	97	111	111
119	125	0	0	296	296	278	278	97	97	119	127
121	131	0	0	296	296	278	278	97	97	95	111
121	131	0	0	296	296	278	278	97	97	95	111
119	131	0	0	296	296	278	278	97	97	119	121
123	131	0	0	296	296	278	278	0	0	111	127
121	123	0	0	296	296	278	278	97	97	111	111
119	131	0	0	296	296	278	278	97	97	95	111
131	131	0	0	296	296	278	278	97	97	119	121
121	121	0	0	296	296	278	278	97	97	95	115
119	121	0	0	296	296	278	278	97	97	119	121
115	123	0	0	296	296	278	278	97	97	119	121
119	131	0	0	296	296	278	278	97	97	95	119
121	131	0	0	296	296	278	278	97	97	119	119
125	131	0	0	296	296	278	278	97	97	111	127

125	131	0	0	296	296	278	278	97	97	113	119
125	131	0	0	296	296	278	278	97	97	111	119
121	123	0	0	296	296	278	278	97	97	95	95
131	131	0	0	296	296	278	278	97	97	111	111
119	131	0	0	296	296	278	278	97	97	119	127
119	131	0	0	296	296	278	278	97	97	95	95
123	131	0	0	296	299	278	278	97	97	95	119
121	131	0	0	296	296	278	278	97	97	95	95
115	131	0	0	296	296	278	278	97	97	111	111
121	131	0	0	296	296	278	278	97	97	95	119
123	125	0	0	296	296	278	278	97	97	95	119
119	121	0	0	293	299	278	278	97	97	119	125
121	121	0	0	293	296	278	278	97	97	95	111
129	129	0	0	293	287	278	278	97	97	95	119
119	119	0	0	296	296	278	278	97	97	95	111
127	NA	0	NA	0	NA	0	NA	97	NA	107	NA
115	NA	0	NA	0	NA	0	NA	97	NA	119	NA
125	NA	0	NA	0	NA	0	NA	97	NA	119	NA
121	NA	0	NA	0	NA	0	NA	97	NA	107	NA
125	NA	0	NA	0	NA	0	NA	97	NA	95	NA
131	NA	0	NA	0	NA	0	NA	97	NA	95	NA
131	NA	0	NA	0	NA	0	NA	97	NA	95	NA
119	NA	0	NA	296	NA	278	NA	97	NA	95	NA
113	NA	0	NA	296	NA	278	NA	97	NA	111	NA
113	NA	0	NA	296	NA	278	NA	97	NA	95	NA
131	NA	0	NA	0	NA	0	NA	97	NA	95	NA
131	NA	0	NA	0	NA	0	NA	97	NA	111	NA
119	NA	0	NA	296	NA	278	NA	97	NA	95	NA
121	NA	0	NA	0	NA	0	NA	0	NA	111	NA
119	NA	0	NA	0	NA	0	NA	97	NA	111	NA
119	NA	0	NA	0	NA	0	NA	97	NA	111	NA
131	NA	0	NA	296	NA	278	NA	97	NA	111	NA
119	NA	0	NA	0	NA	0	NA	97	NA	115	NA
123	NA	0	NA	0	NA	0	NA	0	NA	95	NA
125	NA	0	NA	296	NA	0	NA	97	NA	95	NA
131	NA	0	NA	0	NA	0	NA	97	NA	111	NA
119	NA	0	NA	0	NA	0	NA	97	NA	111	NA
139	NA	0	NA	0	NA	0	NA	97	NA	119	NA
119	NA	0	NA	0	NA	0	NA	97	NA	111	NA
125	NA	0	NA	296	NA	278	NA	97	NA	111	NA
119	NA	0	NA	296	NA	278	NA	97	NA	111	NA
119	NA	0	NA	0	NA	0	NA	97	NA	95	NA
119	NA	0	NA	0	NA	0	NA	97	NA	95	NA
131	NA	0	NA	0	NA	0	NA	0	NA	0	NA
131	NA	0	NA	293	NA	278	NA	97	NA	119	NA
131	NA	0	NA	293	NA	278	NA	97	NA	119	NA
119	NA	0	NA	296	NA	278	NA	97	NA	119	NA
119	NA	0	NA	296	NA	278	NA	97	NA	119	NA
119	NA	0	NA	291	NA	278	NA	97	NA	119	NA
119	NA	0	NA	296	NA	278	NA	97	NA	95	NA

119	NA	0	NA	296	NA	278	NA	97	NA	95	NA
131	NA	0	NA	296	NA	278	NA	97	NA	119	NA
119	NA	0	NA	296	NA	278	NA	97	NA	95	NA
131	NA	0	NA	296	NA	278	NA	97	NA	95	NA
119	NA	0	NA	296	NA	278	NA	97	NA	95	NA
119	NA	0	NA	296	NA	278	NA	97	NA	119	NA
131	NA	0	NA	296	NA	278	NA	97	NA	95	NA
119	NA	227	NA	296	NA	278	NA	97	NA	111	NA
119	NA	0	NA	296	NA	278	NA	97	NA	121	NA
119	NA	0	NA	291	NA	278	NA	97	NA	111	NA
117	NA	0	NA	296	NA	278	NA	97	NA	111	NA
0	NA	0	NA	0	NA	0	NA	0	NA	0	NA
133	NA	0	NA	296	NA	278	NA	97	NA	119	NA
119	NA	0	NA	296	NA	278	NA	97	NA	111	NA
133	NA	0	NA	296	NA	278	NA	97	NA	111	NA
121	NA	0	NA	296	NA	278	NA	97	NA	113	NA
131	NA	0	NA	296	NA	278	NA	97	NA	111	NA
119	NA	0	NA	296	NA	278	NA	97	NA	111	NA
119	NA	0	NA	296	NA	278	NA	97	NA	119	NA
121	NA	0	NA	296	NA	278	NA	97	NA	95	NA
113	NA	0	NA	291	NA	278	NA	97	NA	111	NA
119	NA	0	NA	296	NA	278	NA	97	NA	111	NA
119	NA	0	NA	293	NA	278	NA	97	NA	119	NA
127	NA	0	NA	296	NA	278	NA	97	NA	111	NA
119	NA	0	NA	296	NA	278	NA	97	NA	111	NA
113	NA	0	NA	299	NA	278	NA	97	NA	111	NA
119	NA	0	NA	296	NA	278	NA	97	NA	111	NA
115	NA	0	NA	296	NA	278	NA	97	NA	119	NA
119	NA	0	NA	296	NA	278	NA	97	NA	119	NA
125	NA	0	NA	296	NA	278	NA	97	NA	119	NA
119	NA	0	NA	296	NA	278	NA	97	NA	119	NA
131	NA	0	NA	296	NA	278	NA	97	NA	111	NA
121	NA	0	NA	296	NA	278	NA	97	NA	95	NA
121	NA	0	NA	296	NA	278	NA	97	NA	95	NA
119	NA	0	NA	296	NA	278	NA	97	NA	95	NA
123	NA	0	NA	296	NA	278	NA	97	NA	125	NA
123	98	0	187	296	281	278	313	0	108	115	0
123	98	0	191	296	281	278	313	0	122	115	0
123	0	0	191	296	281	278	313	97	114	115	0
123	98	0	187	0	0	0	0	97	106	115	0
123	98	0	191	0	0	0	0	97	106	115	95
123	0	0	198	296	281	278	311	97	112	115	0
123	98	0	198	296	281	278	315	97	116	115	0
123	0	0	187	296	281	278	0	97	114	115	0
123	98	0	198	296	281	278	311	97	112	115	0
123	98	0	189	296	287	278	319	97	108	115	0
123	98	0	179	296	281	278	321	97	110	115	0
123	98	0	191	296	281	278	313	97	110	115	0
123	98	0	191	296	281	278	313	97	110	115	0
123	98	0	198	296	281	278	313	97	116	115	0

123	98	0	191	296	281	278	315	97	116	115	0
123	98	0	191	296	287	278	315	97	116	115	0
123	0	0	191	296	281	278	313	97	114	115	0
123	98	0	214	296	0	278	319	97	116	115	0
123	98	0	0	296	281	278	313	97	0	115	0
123	98	0	0	0	0	0	0	97	116	115	0
123	98	0	0	0	0	0	0	97	112	115	0
123	98	0	203	296	281	278	315	97	112	115	0
123	98	0	187	296	281	278	313	97	116	115	0
123	98	0	0	0	0	0	0	97	108	115	0
123	98	0	0	0	0	0	0	97	108	115	0
123	98	0	191	296	287	0	0	97	108	115	0
123	98	0	203	0	0	0	0	97	106	115	0
98	98	187	0	281	281	0	0	106	0	0	0
98	98	187	191	281	281	313	313	106	114	0	0
98	98	191	0	281	287	303	313	110	110	0	0
98	98	187	0	287	296	313	315	114	0	0	0
98	98	187	0	287	296	307	313	106	114	0	0
98	98	187	0	281	281	307	313	93	106	0	0
98	98	187	187	287	296	313	313	93	106	0	0
98	98	187	191	281	296	307	313	93	106	0	0
98	98	187	195	281	281	313	317	106	0	0	0
98	98	191	203	275	281	313	317	110	114	0	0
98	98	191	203	281	281	313	317	106	108	0	0
98	98	191	0	281	281	311	313	114	137	0	0
98	98	187	191	281	281	313	315	114	137	0	0
98	98	187	187	281	287	311	313	106	108	0	0
98	98	191	207	281	281	311	313	106	114	0	0
98	98	189	191	281	287	313	313	106	106	0	0
98	98	191	198	281	281	0	0	108	116	0	0
0	0	187	203	281	287	313	321	116	0	0	0
98	98	195	214	281	281	313	317	106	106	0	0
98	98	191	203	281	281	315	321	97	116	0	0
98	98	187	198	281	281	303	313	112	114	0	0
98	98	187	187	281	281	315	317	108	116	0	0
98	98	187	198	281	281	303	315	114	116	0	0
98	98	191	0	287	287	307	313	112	118	0	0
0	0	191	0	281	281	313	319	108	114	0	0
98	98	189	203	0	0	313	313	125	140	0	0
98	98	187	195	281	281	311	313	106	137	0	0
98	98	189	189	0	0	0	0	106	122	0	0
98	98	191	207	281	281	311	313	118	118	0	0
98	98	207	207	281	287	313	313	118	118	0	0
98	98	195	207	281	281	313	313	116	122	0	0
98	98	212	212	281	281	313	313	108	106	0	0
0	0	191	0	281	287	307	313	114	118	0	0
98	98	191	191	281	287	313	315	106	106	0	0
98	98	191	203	281	287	313	313	108	110	0	0
98	98	203	0	281	281	313	319	114	0	0	0
98	98	191	191	281	287	311	313	108	116	0	0

98	98	0	0	281	281	313	313	108	114	0	0
98	98	191	212	281	281	307	313	106	114	0	0
98	98	191	191	281	287	311	313	108	106	0	0
98	98	191	191	281	281	317	317	116	116	0	0
98	98	212	212	281	287	313	317	108	0	0	0
98	98	0	0	279	281	298	311	83	83	0	0

RICA18		Rrid135A	
Allele1	Allele2	Allele1	Allele2
177	186	0	0
177	181	236	236
177	181	0	0
177	181	0	0
177	181	236	236
0	0	0	0
181	181	0	0
181	181	0	0
177	186	236	236
181	181	0	0
186	186	0	0
177	186	236	236
186	186	0	0
177	181	0	0
177	179	236	236
181	186	236	236
177	181	0	0
177	177	0	0
181	186	0	0
0	0	0	0
177	184	0	0
177	186	0	0
181	181	0	0
188	188	0	0
0	0	0	0
0	0	0	0
177	188	0	0
181	186	0	0
177	188	236	236
177	181	0	0
181	186	0	0
177	177	0	0
177	177	236	236
181	186	236	236
186	186	236	236
186	186	236	236
0	0	0	0
177	181	236	236
186	188	236	236
0	0	0	0
181	186	236	236
0	0	0	0
181	197	236	236

184	184	0	0
181	186	236	236
179	181	199	0
181	186	236	236
184	186	236	236
177	181	236	236
179	188	236	236
181	195	236	236
0	0	0	0
181	202	0	0
184	184	236	236
181	186	236	236
177	202	236	236
181	181	199	0
177	181	236	236
181	181	236	236
177	181	0	0
181	186	236	236
181	186	236	236
177	181	236	236
184	186	0	0
186	190	0	0
175	190	0	0
179	181	0	0
179	181	0	0
181	186	0	0
186	186	0	0
184	186	0	0
177	190	0	0
184	186	0	0
186	190	0	0
181	186	0	0
190	195	0	0
175	179	0	0
181	188	0	0
184	188	0	0
186	190	0	0
186	190	0	0
179	186	0	0
177	195	0	0
177	186	0	0
184	184	0	0
186	190	0	0
186	195	0	0
186	190	0	0
175	181	0	0
177	177	0	0
181	186	0	0
179	186	0	0
184	190	0	0

181	186	0	0
181	195	0	0
181	181	0	0
177	181	0	0
181	186	0	0
181	186	0	0
186	186	0	0
181	181	0	0
181	190	0	0
181	186	0	0
181	181	0	0
181	184	0	0
181	184	0	0
186	186	0	0
186	186	0	0
181	NA	0	NA
181	NA	0	NA
186	NA	0	NA
0	NA	0	NA
186	NA	0	NA
181	NA	0	NA
186	NA	0	NA
181	NA	0	NA
181	NA	0	NA
186	NA	236	NA
181	NA	0	NA
181	NA	0	NA
186	NA	0	NA
181	NA	0	NA
0	NA	0	NA
0	NA	0	NA
177	NA	236	NA
186	NA	0	NA
0	NA	0	NA
181	NA	0	NA
0	NA	0	NA
0	NA	0	NA
186	NA	0	NA
0	NA	0	NA
186	NA	236	NA
195	NA	0	NA
0	NA	0	NA
181	NA	0	NA
0	NA	0	NA
181	NA	236	NA
181	NA	236	NA
181	NA	236	NA
181	NA	236	NA
177	NA	236	NA
181	NA	0	NA

181	NA	236	NA
181	NA	0	NA
177	NA	236	NA
186	NA	236	NA
177	NA	0	NA
177	NA	236	NA
177	NA	236	NA
177	NA	236	NA
186	NA	236	NA
186	NA	236	NA
186	NA	0	NA
0	NA	0	NA
181	NA	236	NA
177	NA	236	NA
184	NA	236	NA
175	NA	0	NA
179	NA	236	NA
186	NA	0	NA
181	NA	0	NA
186	NA	0	NA
181	NA	0	NA
195	NA	236	NA
173	NA	236	NA
186	NA	0	NA
177	NA	236	NA
186	NA	0	NA
186	NA	0	NA
177	NA	236	NA
195	NA	236	NA
181	NA	0	NA
169	NA	0	NA
177	NA	236	NA
181	NA	236	NA
181	NA	236	NA
177	NA	236	NA
181	NA	236	NA
186	0	0	203
186	0	0	203
186	0	0	203
186	0	0	0
186	0	0	0
186	0	236	183
186	0	236	199
186	0	236	203
186	0	236	183
186	0	236	203
186	0	236	199
186	0	236	205
186	0	236	169
186	0	236	199

186	0	236	0
186	0	236	0
186	0	236	203
186	0	236	0
186	0	0	199
186	0	0	0
186	0	0	0
186	0	236	169
186	0	0	199
186	0	0	0
186	0	0	203
186	0	0	203
186	0	0	203
0	0	169	199
0	0	169	169
0	0	169	183
0	0	169	199
0	0	169	169
0	0	169	169
0	0	169	203
0	0	169	203
0	0	199	203
0	0	169	203
0	0	169	203
0	0	203	203
0	0	0	0
0	0	169	199
0	0	169	203
0	0	169	203
0	0	199	203
0	0	169	203
0	0	203	203
0	0	169	199
0	0	199	203
0	0	199	205
0	0	199	203
0	0	0	0
0	0	169	203
0	0	0	0
0	0	199	199
0	0	203	203
0	0	199	203
0	0	203	203
0	0	203	203
0	0	203	203
0	0	199	205
0	0	203	203
0	0	199	203
0	0	199	199
0	0	199	199

all

0	0	169	169
0	0	203	203
0	0	199	203
0	0	199	203
0	0	205	205
0	0	169	185
