# Supplementary material for: All-male hybrids of a tetrapod Pelophylax esculentus share its origin and genetics of maintenance
Source: Biol Sex Differ. 2018 Apr 2;9:13. doi: 10.1186/s13293-018-0172-z (PMC5880063; doi:10.1186/s13293-018-0172-z)
Supplement: Supplementary file 3 — Table S3. Allozyme data file for the six loci used in the study. (PDF 514 kb) [file 13293_2018_172_MOESM3_ESM.pdf]

Tab. S3: Allozyme data file for the six loci used in the study.

Description: This Table lists the alleles found in each sampled individual (specified by a respective Sample ID) on the basis of six allozyme loci from somatic (soma) and gonadal (gonad) tissues. Grey colour indicates introgressed alleles.

**Sample ID**	**Taxon**	**Allozyme locus**										
		Ldh B		***GPI***		***aaT***		***PGM***		***G3PDH***	***6-PGD***	
		**soma**	**gonad**	**soma**	**gonad**	**soma**	**gonad**	**soma**	**gonad**	**soma**	**soma**	**gonad**
BI-11-RR	RR	aa	aa	aa	aa	aa	aa	cc	cc	aa	cc	cc
Da-1-RR	RR	ac	ac	aa		aa	aa	cc		aa	bb	
Da-2-RR	RR	aa		bb		aa		cc		aa	bb	
Da-3-RR	RR	ac	ac	ab	ab	aa	aa	cc		aa	bc	
Da-4-RR	RR	aa		aa		aa		cc		aa	bc	
Da-5-RR	RR	ac	ac	ab	ab	aa	aa	cc		ab	bc	bc
Da-6-RR	RR	aa	aa	aa	aa	aa	aa	cc		aa	bb	bb
Da-7-RR	RR	aa		ab		aa		cc	cc	aa	bc	
Da-8-RR	RR	ac	ac	aa	aa	aa	aa	cc	cc	aa	bb	
DB-2-RR	RR	ac	ac	ab	ab	aa	aa	cc	cc	bb	bb	
DB-3-RR	RR	aa	aa	bb	bb	aa	aa	cc	cc	aa	bb	
DB-6-RR	RR	ac		ab		aa		cc		aa	bc	
DB-7-RR	RR	aa		aa		aa		cc		aa	bb	
DB-8-RR	RR	aa		aa		aa		aa		aa	bb	
DB-9-RR	RR	ac		aa		aa		cc		aa	bc	
OS-13-RR	RR	aa		aa		aa		cc		aa	cc	ab
OS-1-RR	RR	aa	aa	bb	bb	aa	aa	cc		aa	bc	
OS-2-RR	RR	cc	cc	ab	ab	aa	aa	bb	bb	aa	bc	bc
OS-3-RR	RR	aa	aa	ab	ab	aa	aa	cc	cc	aa	bb	bb
OS-4-RR	RR	cc	cc	ab	ab	aa	aa	cc	cc	aa	bb	bb
OS-5-RR	RR	aa	aa	ab	ab	aa	aa	cc	cc	aa	bc	bc
OS-7-RR	RR	aa	aa	aa	aa	aa	aa	cc	cc	aa	bb	bb
OS-8-RR	RR	aa		ab		aa		cc		aa	bc	
OS-9-RR	RR	cc		aa		aa		bc		aa	bc	ab
PO-11-RR	RR	aa	aa	ab	ab	aa	aa	cc	cc	aa	bb	bb
PO-12-RR	RR	aa	aa	ab	ab	aa	aa	cc	cc	aa	bb	bb

PO-13-RR	RR	ac	ac	aa	aa	aa	aa	cc	cc	aa	bc	bc
PO-4-RR	RR	aa		bb		aa		cc		aa	bb	
PO-5-RR	RR	cc		ab		aa		ac		aa	bc	
BI-6-RL	LL	bd	bd	bb	bb	bb	bb	bb	bb	bb	aa	aa
BT-10-RL	LL	dd		bb		bb		bb		bb	aa	
BT-4-RL	LL	dd	dd	bb	bb	bb	bb	bb	bb	bb	aa	aa
BT-5-RL	LL	dd		bb		bb		bb		bb	aa	
BT-6-RL	LL	bd		bb		bb		bb		bb	aa	
BT-7-RL	LL	dd		bb		bb		bb		bb	aa	
BT-8-RL	LL	dd		bb		bb		bb		bb	aa	
BT-9-RL	LL	dd		bb		bb		bb		bb	aa	
CT-10-RL	LL	bd		bb		bb		bb		bb	aa	
CT-11-RL	LL	bd		bb		bb		bb		bb	aa	
CT-1-RL	LL	bd	bd	bb	bb	bb	bb	bb	bb	bb	aa	aa
CT-2-RL	LL	dd		bb		bb		bb		bb	aa	
CT-3-RL	LL	bd	bd	bb	bb	bb	bb	bb	bb	bb	aa	
CT-4-RL	LL	bd	bd	bb	bb	bb	bb	bb	bb	bb	aa	
CT-5-RL	LL	bd	bd	bb	bb	bb	bb	bb	bb	bb	aa	aa
CT-6-RL	LL	bd	bd	bb	bb	bb	bb	bb	bb	bb	aa	aa
CT-7-RL	LL	dd		bb		bb		bb		bb	aa	
CT-8-RL	LL	dd		bb		bb		bb		bb	aa	
DS-1-RL	LL	dd		bb		bb		bb		00	aa	
DS-2-RL	LL	bd	bd	bb	bb	bb	bb	bb	bb	bb	aa	aa
HB-10-RL	LL	dd	dd	bb	bb	bb	bb	bb	bb	bb	aa	aa
HB-11-RL	LL	dd		bb		bb		bb		bb	aa	
HB-12-RL	LL		dd	bb	bb	bb	bb	bb	bb	bb	aa	aa
HB-13-RL	LL	bd	bd	bb	bb	bb	bb	bb	bb	bb	aa	aa
HB-14-RL	LL	dd		bb		bb		bb		bb	aa	
HB-15-RL	LL	dd		bb		bb		bb		00	aa	
HB-1-RL	LL	dd		bb		bb		bb		00	aa	
HB-2-RL	LL	bd	bd	bb	bb	bb	bb	bb	bb	00	aa	aa
HB-3-RL	LL	bd	bd	bb	bb	bb	bb	bb	bb	bb	aa	aa
HB-4-RL	LL	dd		bb		bb		bb		bb	aa	

HB-5-RL	LL	dd		bb		bb		bb		bb	aa	
HB-6-RL	LL	bd	bd	bb	bb	bb	bb	bb	bb	bb	aa	aa
HB-7-RL	LL	bd	bd	bb	bb	bb	bb	bb	bb	bb	aa	aa
HB-8-RL	LL	bb		bb		bb		bb		bb	aa	
HD-12-RL	LL	bd	bd	bb	bb	bb	bb	bb		bb	aa	
HD-13-RL	LL	bd		bb		bb		bb		bb	aa	
HD-14-RL	LL	bd	bd	bb	bb	bb	bb	bb		bb	aa	
HD-15-RL	LL	bd	bd	bb	bb	bb	bb	bb		bb	aa	
HD-4-RL	LL	bd	bd	bb	bb	bb	bb	bb		bb	aa	
HD-6-RL	LL	bd		bb		bb		bb		bb	aa	
HD-9-RL	LL	bd		bb		bb		bb		bb	aa	
Ka-13-RL	LL	bd		bb		bb		bb		bb	aa	
Ka-9-RL	LL	dd	dd	bb	bb	bb	bb	bb	bb	bb	aa	
LO-9-RL	LL	dd	dd	bb	bb	bb	bb	bb		bb	aa	
PR-11-RL	LL	bd	bd	bb	bb	bb		bb		bb	aa	
PR-2-RL	LL	bd	bd	bb	bb	bb	bb	bb	bb	bb	aa	
PR-3-RL	LL	bd	bd	bb	bb	bb	bb	bb		bb	aa	aa
PR-4-RL	LL	dd		bb		bb		bb		bb	aa	
PR-5-RL	LL	dd		bb		bb		bb		bb	aa	
PR-6-RL	LL	dd		bb		bb		bb		bb	aa	
PR-7-RL	LL	bd	bd	bb	bb	bb	bb	ab	ab	bb	aa	
PR-8-RL	LL	bb	bb	bb	bb	bb	bb	bb	bb	bb	aa	
TR-10-RL	LL	bd	bd	bb	bb	bb	bb	bb	bb	bb	aa	
TR-11-RL	LL	bd	bd	bb	bb	bb	bb	bb	bb	bb	aa	aa
TR-12-RL	LL	bb		bb		bb		bb		bb	aa	
TR-13-RL	LL	bb		bb		bb		bb		bb	aa	
TR-14-RL	LL	dd		bb		bb		bb		bb	aa	
TR-15-RL	LL	bd	bd	bb	bb	bb	bb	bb	bb	bb	aa	
TR-3-RL	LL	dd		bb		bb		bb		bb	aa	
TR-4-RL	LL	bb		bb		bb		bb		bb	aa	
TR-5-RL	LL	bd	bd	bb		bb	bb	bb		bb	aa	
TR-8-RL	LL	dd		bb		bb		bb		bb	aa	
TR-9-RL	LL	bd	bd	bb		bb	bb	bb	bb	bb	aa	

BI-7-RL	RL	cd	dd	bb	bb	ab	bb	bc	bb	ab	ab	bb
BI-8-RL	RL	cd	dd	ab	bb	ab	bb	bc	bb	ab	ab	bb
Da-10-RL	RL	ad		ab		ab		bb		ab	ab	
Da-11-RL	RL	ad		bb		ab		bb		ab	ab	
Da-12-RL	RL	ad		ab		ab		bc		ab	ab	
Da-9-RL	RL	ad		bb		ab		bb		ab	ac	
DB-10-RL	RL	cd		ab		ab		bc		ab	ac	
DB-1-RL	RL	ad	dd	ab	bb	ab	bb	bc		ab	ab	
DB-4-RL	RL	ad		ab		ab		bb		ab	ab	
DB-5-RL	RL	ad		ab		ab		cc		ab	ab	
OS-10-RL	RL	cd		bb		ab		bc		ab	bc	ab
OS-11-RL	RL	cd		bb		ab		bc		ab	bb	
OS-12-RL	RL	cd		bb		ab		bc		ab	bb	ab
OS-14-RL	RL	cd		ab		ab		bc		bb	bb	
OS-6-RL	RL	cd	dd	ab	ab	ab	bb	bc		ab	ac	ac
PO-10-RL	RL	cd		ab		ab		ab		ab	bb	
PO-14-RL	RL	cd	dd	ab	bb	ab	bb	ab	bb	ab	ac	aa
PO-15-RL	RL	ad	dd	ab	bb	ab	bb	ab		ab	bb	
PO-1-RL	RL	cd	dd	ab	bb	ab	bb	bc		ab	ac	
PO-2-RL	RL	cd		ab		ab		bc		ab	bb	
PO-3-RL	RL	cd				ab		bc		ab	ab	
PO-7-RL	RL	cd		ab		ab		bc		ab	bb	
PO-8-RL	RL	cd		ab		ab		bc		ab	ac	
PO-9-RL	RL	cd	dd	ab	bb	ab	bb	bc	bb	ab	bb	
BI-7-RL	RL	cd	dd	bb	bb	ab	bb	bc	bb	ab	ab	bb
BI-8-RL	RL	cd	dd	ab	bb	ab	bb	bc	bb	ab	ab	bb
Da-10-RL	RL	ad		ab		ab		bb		ab	ab	
Da-11-RL	RL	ad		bb		ab		bb		ab	ab	
Da-12-RL	RL	ad		ab		ab		bc		ab	ab	
Da-9-RL	RL	ad		bb		ab		bb		ab	ac	
DB-10-RL	RL	cd		ab		ab		bc		ab	ac	
DB-1-RL	RL	ad	dd	ab	bb	ab	bb	bc		ab	ab	
DB-4-RL	RL	ad		ab		ab		bb		ab	ab	

DB-5-RL	RL	ad		ab		ab		cc		ab	ab	
OS-10-RL	RL	cd		bb		ab		bc		ab	bc	ab
OS-11-RL	RL	cd		bb		ab		bc		ab	bb	
OS-12-RL	RL	cd		bb		ab		bc		ab	bb	ab
OS-14-RL	RL	cd		ab		ab		bc		bb	bb	
OS-6-RL	RL	cd	dd	ab	ab	ab	bb	bc		ab	ac	ac
PO-10-RL	RL	cd		ab		ab		ab		ab	bb	
PO-14-RL	RL	cd	dd	ab	bb	ab	bb	ab	bb	ab	ac	aa
PO-15-RL	RL	ad	dd	ab	bb	ab	bb	ab		ab	bb	
PO-1-RL	RL	cd	dd	ab	bb	ab	bb	bc		ab	ac	
PO-2-RL	RL	cd		ab		ab		bc		ab	bb	
PO-3-RL	RL	cd				ab		bc		ab	ab	
PO-7-RL	RL	cd		ab		ab		bc		ab	bb	
PO-8-RL	RL	cd		ab		ab		bc		ab	ac	
PO-9-RL	RL	cd	dd	ab	bb	ab	bb	bc	bb	ab	bb	
BI-10-RL	RL	cd	cc	ab	aa	ab	aa	bc	cc	ab	bb	bb
BI-12-RL	RL	cd	cc	ab	aa	ab	aa	bc	cc	ab	bb	bb
BI-2-RL	RL	cd		ab		ab		bc		ab	bb	
BI-3-RL	RL	cd		ab		ab		bc		ab	ab	
BI-4-RL	RL	cd	cc	ab	aa	ab	aa	bc	cc	ab	bb	bb
BI-5-RL	RL	bc	cc	ab	aa	ab	aa	bc	cc	ab	ab	bb
BI-9-RL	RL	cd	cc	ab	aa	ab	aa	bc	cc	ab	ab	bb
BT-1-RL	RL	bc	cc	ab	aa	ab	aa	bc	cc	ab	ab	bb
BT-2-RL	RL	cd		ab		ab		bc		ab	ab	
BT-3-RL	RL	cd	cc	ab	aa	ab	aa	bc	cc	ab	ab	bb
CT-12-RL	RL	ad	aa	bb	bb	ab	aa	bc	cc	ab	ab	bb
CT-13-RL	RL	cd	cc	ab	aa	ab	aa	bc	cc	ab	ab	bb
CT-14-RL	RL	ab	aa	bb	bb	ab	aa	bc	cc	ab	ab	bb
CT-15-RL	RL	ac	cc	ab	aa	ab	aa	bc	cc	ab	ab	bb
CT-9-RL	RL	cd		ab		ab		bc		ab	ab	
DS-10-RL	RL	bc	cc	ab	aa	ab	aa	bc	cc		ab	
DS-11-RL	RL	bc	cc	ab	aa	ab	aa	bc	cc	ab	ab	
DS-12-RL	RL	cd	cc	ab	aa	ab	aa	bc	cc		ab	

DS-13-RL	RL	cd	cc	ab	aa	ab	aa	bc	cc		ab	
DS-14-RL	RL	cd	cc	ab	aa	ab	aa	bc	cc		ab	
DS-15-RL	RL	bc	cc	ab	aa	ab	aa	bc	cc		ab	
DS-3-RL	RL	bc	cc	ab	aa	ab	aa	bc	cc		bb	ab
DS-4-RL	RL	cd	cc	ab	aa	ab	aa	bc	cc		ab	
DS-5-RL	RL	bc	cc	ab	aa	ab	aa	bc	cc	ab	ab	
DS-6-RL	RL	bc	cc	ab	aa	ab	aa	bc	cc	ab	ab	
DS-7-RL	RL	bc	cc	ab	aa	ab	aa	bc	cc	ab	ab	
DS-8-RL	RL	bc	cc	ab	aa	ab	aa	bc	cc		ab	
DS-9-RL	RL	bc	cc	ab	aa	ab	aa	bc	cc	ab	ab	
HB-9-RL	RL	cd	cc	ab	aa	ab	aa	bc	cc	ab	bc	bb
HD-10-RL	RL	cd		ab		ab		bc		ab	ab	
HD-11-RL	RL	cd		ab		ab		bc			ab	
HD-1-RL	RL	bc	cc	ab	aa	ab	aa	bc	cc	ab	bb	cc
HD-2-RL	RL	bc	cc	ab	aa	ab	aa	bc	cc	ab	ab	bb
HD-3-RL	RL	cd	cc	ab	aa	ab	aa	bc			ab	cc
HD-5-RL	RL	bc		ab		ab		bc		ab	bb	
HD-7-RL	RL	cd		aa		ab		bc		ab	ab	
HD-8-RL	RL	cd	cc	ab	aa	ab	aa	bc		ab	ab	
Ka-10-RL	RL	cd		ab		ab		cc		ab	ab	
Ka-11-RL	RL	bc		ab		ab		bc		ab	ab	
Ka-12-RL	RL	cd		ab		ab		bc		ab	ab	
Ka-14-RL	RL	cd		ab		ab		bc		ab	ab	
Ka-15-RL	RL	bc	cc	ab	aa	ab	aa	bc	cc	ab	ab	bb
Ka-1-RL	RL	cd	cc	ab	aa	ab	aa	bc	cc	ab	ab	
Ka-2-RL	RL	bc	cc	ab	aa	ab	aa	bc	cc	ab	ab	
Ka-3-RL	RL	cd	cc	ab	aa	ab	aa	bc	cc	ab	ab	
Ka-4-RL	RL	cd	cc	ab	aa	ab	aa	bc	cc	ab	ab	
Ka-5-RL	RL	cd	cc	ab	aa	ab	aa	bc	cc	ab	ab	
Ka-6-RL	RL	cd	cc	ab	aa	ab	aa	bc		ab	ab	
Ka-7-RL	RL	cd	cc	ab	aa	ab	aa	bc	cc	ab	ab	
Ka-8-RL	RL	cd	cc	ab	aa	ab	aa	bc	cc	ab	ab	bb
LO-10-RL	RL	ab		ab		ab		bc		ab	bb	

LO-11-RL	RL	cd		ab		ab		bc		ab	bb	
LO-12-RL	RL	cd	cc	ab	aa	ab	aa	bc	cc	ab	bb	bb
LO-13-RL	RL	bc		ab		ab		bc		ab	bb	
LO-14-RL	RL	cd		bb		ab		bc		ab	bb	
LO-15-RL	RL	cd	cc	ab	aa	ab	aa	bc	cc	ab	bb	bb
LO-1-RL	RL	cd	cc	ab	aa	ab	aa	bc	cc	ab	bb	bb
LO-2-RL	RL	cd	cc	ab	aa	ab	aa	bc	cc	ab	bb	bb
LO-3-RL	RL	cd	cc	ab	aa	ab	aa	bc	cc	ab	bb	bb
LO-4-RL	RL	bc	cc	ab	aa	ab	aa	bc	cc	ab	bb	bb
LO-5-RL	RL	cd	cc	ab	aa	ab	aa	bc	cc	ab	bb	
LO-6-RL	RL	cd	cc	ab	aa	ab	aa	bc	cc	ab	bb	bb
LO-7-RL	RL	cd	cc	ab	aa	ab	aa	bc	cc	ab	bb	bb
LO-8-RL	RL	cd	cc	ab	aa	ab	aa	bc	cc	ab	bb	bb
PR-10-RL	RL	bc		ab		ab		bc			ab	
PR-1-RL	RL	bc	cc	ab	aa	ab	aa	bc	cc	ab	ab	bb
PR-9-RL	RL	cd		ab		ab		bc		ab	ab	
TR-1-RL	RL	cd	cc	ab	aa	ab	aa	bc		ab	bb	bb
TR-2-RL	RL	cd	cc	ab	aa	ab		bc		ab	bb	
TR-6-RL	RL	bc	cc	ab		ab	aa	bc	cc	ab	bb	bb
TR-7-RL	RL	cd	cc	ab	aa	ab	aa	bc	cc	ab	bb	bb

Notes: RR, *P. ridibundus;* RL, *P. esculentus;* LL, *P. lessonae*
