# Supplementary material for: All-male hybrids of a tetrapod Pelophylax esculentus share its origin and genetics of maintenance
Source: Biol Sex Differ. 2018 Apr 2;9:13. doi: 10.1186/s13293-018-0172-z (PMC5880063; doi:10.1186/s13293-018-0172-z)
Supplement: Supplementary file 4 — Table S4. Species-specificity of microsatellite alleles used in this study. (PDF 259 kb) [file 13293_2018_172_MOESM4_ESM.pdf]

Tab. S4: Species-specificity of microsatellite alleles used in this study.
Description: This table lists a number of *P. lessonae* and *P. ridibundus* species-specific alleles that amplified in 17 microsatellite loci at analysed Pelophylax individuals. One allele that amplified at both species is also mentioned.

**Locus**	**L specific alleles**	**R specific alleles**	**Non-specific allele**
RICA1b6	78/80	74/85/92/94/98	83
RICA1b5	113/118	132/134/136/138	
Ga1a19	195	201/205/209/217/223/239/243/247/249/253/255/259	
RICA5	247/252/256/258/260/262/264	232/234/236/248/250	
Res16	121/133/152	115/117/119/123/127	
Res20	102/104/108/110/112/114/116/118/120/122/124/126/128/131/142/144/146	__	
RICA2a34	112/123/130/136/138/140/143/145/147/150/152/154/156/162/164	106/110	
Re2Caga3	__	169/192/196/200/204/208/212/220/227/231/235/255	
Res22	__	83/87/98/104/106/108/110/114/116/124/127/129	
Ga1a23	113/115/117/119/121/123/125/127/129/131/133/135/139/141	98	
Rrid169A	__	179/187/189/191/193/195/198/203/207/212/214/227/234	
Rrid013A	291/293/296/299	281/287	
Rrid059A	278	303/307/311/313/315/317/319/321	
Re1Caga10	97	93/106/108/110/112/114/116/118/122/125/137/140	
RICA1a27	95/111/113/115/117/119/121/125/127	__	

RICA18	175/177/179/181/184/186/188/190/195/197/202	__
Rrid135A	236	169/183/199/203/205

Notes: L, *P. lessonae* ; R, *P. ridibundus; Non-specific allele, allele that amplified at both species.*
