# Supplementary material for: All-male hybrids of a tetrapod Pelophylax esculentus share its origin and genetics of maintenance
Source: Biol Sex Differ. 2018 Apr 2;9:13. doi: 10.1186/s13293-018-0172-z (PMC5880063; doi:10.1186/s13293-018-0172-z)
Supplement: Supplementary file 5 — Table S5. Summary statistics of microsatellite alleles found in P. lessonae, P. ridibundus, and P. esculentus from the R-E system. (PDF 317 kb) [file 13293_2018_172_MOESM5_ESM.pdf]

Tab. S5: Summary statistics of microsatellite alleles found in *P. lessonae* , *P. ridibundus,* and *P. esculentus* from the R-E system.
Description: This table lists observed and expected heterozygosity, variance in allele length, number of alleles determined in each population, and frequences of alleles that amplified at 17 microsatellite loci.

**Group**	**Locus name**	**H_{obs}**	**H_{exp}**	**Var**	**NA**	**Frequences of alleles**					
LL	RICA1b6	0,528	0,520	1,646	3	0,333	0,606	0,060			
LL	RICA1b5	0,038	0,037	2,653	3	0,009	0,981	0,009			
LL	Ga1a19	0,009	0,009	0,168	2	0,995	0,005				
LL	RICA5	0,480	0,555	6,649	7	0,010	0,034	0,578	0,010	0,333	0,015
LL	Res16	0,010	0,019	5,270	3	0,990	0,005	0,005			
LL	Res20	0,581	0,836	76,863	17	0,012	0,006	0,036	0,301	0,018	0,018
						0,018	0,084	0,054	0,042	0,006	0,030
LL	RICA2a34	0,804	0,770	62,481	15	0,033	0,005	0,005	0,042	0,019	0,093
						0,065	0,070	0,023	0,005	0,023	0,005
LL	Re2Caga3	0,000	1,000	8,000	2	0,500	0,500				
LL	Res22	0,154	0,257	55,114	3	0,067	0,067	0,867			
LL	Ga1a23	0,765	0,830	38,260	15	0,015	0,020	0,029	0,015	0,284	0,176
						0,020	0,211	0,005	0,005	0,005	0,005
LL	Rrid169A	0,333	0,773	389,295	4	0,333	0,333	0,250	0,083		
LL	Rrid013A	0,180	0,199	1,548	5	0,006	0,011	0,039	0,893	0,051	
LL	Rrid059A	0,000	0,000	0,000	1	1,000					
LL	Re1Caga10	0,039	0,039	4,279	3	0,980	0,010	0,010			
LL	RICA1a27	0,738	0,764	100,737	9	0,252	0,345	0,019	0,019	0,005	0,218
LL	RlCA18	0,750	0,801	23,203	11	0,015	0,165	0,040	0,305	0,075	0,265
						0,005	0,010				
LL	Rrid135A	0,000	0,066	44,860	2	0,033	0,967				
L (RL from R-E)	RlCA1b6	0,000	0,000	0,000	1	1,000					
L (RL from R-E)	RlCA1b5	0,000	0,000	0,000	1	1,000					
L (RL from R-E)	Ga1a19	0,000	0,000	0,000	1	1,000					
L (RL from R-E)	RlCA5	0,000	0,000	0,000	1	1,000					
L (RL from R-E)	Res16	0,000	0,000	0,000	1	1,000					

L (RL from R-E)	Res20	0,000	0,000	0,000	1	1,000					
L (RL from R-E)	RICA2a34	0,000	0,000	0,000	1	1,000					
L (RL from R-E)	Re2Caga3	n.d.	n.d.	n.d.	n.d.						
L (RL from R-E)	Res22	n.d.	n.d.	n.d.	n.d.						
L (RL from R-E)	Ga1a23	0,000	0,000	0,000	1	1,000					
L (RL from R-E)	Rrid169A	n.d.	n.d.	n.d.	n.d.						
L (RL from R-E)	Rrid013A	0,000	0,000	0,000	1	1,000					
L (RL from R-E)	Rrid059A	0,000	0,000	0,000	1	1,000					
L (RL from R-E)	Re1Caga10	0,000	0,000	0,000	1	1,000					
L (RL from R-E)	RICA1a27	0,000	0,000	0,000	1	1,000					
L (RL from R-E)	RlCA18	0,000	0,000	0,000	1	1,000					
L (RL from R-E)	Rrid135A	0,000	0,000	0,000	1	1,000					
RR	RlCA1b6	0,585	0,648	21,403	7	0,012	0,024	0,049	0,195	0,549	0,122
RR	RlCA1b5	0,119	0,115	0,376	4	0,012	0,940	0,036	0,012		
RR	Ga1a19	0,732	0,788	386,581	12	0,366	0,244	0,012	0,061	0,037	0,012
						0,024	0,012	0,012			
RR	RlCA5	0,333	0,575	68,914	6	0,639	0,139	0,028	0,056	0,083	0,056
RR	Res16	0,146	0,746	19,064	6	0,106	0,064	0,106	0,191	0,085	0,447
RR	Res20	n.d.	n.d.	n.d.	n.d.						
RR	RICA2a34	0,225	0,339	2,711	2	0,788	0,213				
RR	Re2Caga3	0,850	0,826	670,430	12	0,363	0,075	0,013	0,088	0,013	0,050
						0,125	0,075	0,013			
RR	Res22	0,619	0,666	144,579	12	0,119	0,036	0,012	0,024	0,036	0,024
						0,012	0,012	0,083			
RR	Ga1a23	0,000	0,000	0,000	1	1,000					
RR	Rrid169A	0,488	0,805	60,968	9	0,246	0,049	0,344	0,066	0,049	0,115
RR	Rrid013A	0,400	0,419	15,579	4	0,013	0,738	0,200	0,050		
RR	Rrid059A	0,744	0,657	11,626	8	0,038	0,077	0,090	0,564	0,077	0,103
RR	Re1Caga10	0,690	0,862	78,551	13	0,042	0,014	0,254	0,155	0,042	0,028
						0,028	0,014	0,042	0,014		
RR	RICA1a27	n.d.	n.d.	n.d.	n.d.						
RR	RlCA18	n.d.	n.d.	n.d.	n.d.						
RR	Rrid135A	0,590	0,700	216,667	5	0,269	0,013	0,256	0,410	0,051	

R (RL from R-E)	RlCA1b6	0,000	0,573	12,969	4	0,074	0,185	0,630	0,111		
R (RL from R-E)	RlCA1b5	0,000	0,157	0,333	3	0,040	0,920	0,040			
R (RL from R-E)	Ga1a19	0,000	0,683	211,440	6	0,520	0,240	0,040	0,080	0,080	0,040
R (RL from R-E)	RlCA5	0,000	0,900	104,000	4	0,400	0,200	0,200	0,200		
R (RL from R-E)	Res16	0,000	0,425	24,250	3	0,188	0,750	0,063			
R (RL from R-E)	Res20	n.d.	n.d.	n.d.	n.d.						
R (RL from R-E)	RICA2a34	0,000	0,479	3,832	2	0,650	0,350				
R (RL from R-E)	Re2Caga3	0,000	0,814	492,470	9	0,409	0,045	0,136	0,045	0,045	0,091
R (RL from R-E)	Res22	0,000	0,493	123,883	5	0,148	0,037	0,074	0,704	0,037	
R (RL from R-E)	Ga1a23	0,000	0,000	0,000	1	1,000					
R (RL from R-E)	Rrid169A	0,000	0,788	53,950	7	0,045	0,182	0,045	0,409	0,182	0,091
R (RL from R-E)	Rrid013A	0,000	0,281	5,053	2	0,842	0,158				
R (RL from R-E)	Rrid059A	0,000	0,712	7,529	5	0,111	0,500	0,222	0,111	0,056	
R (RL from R-E)	Re1Caga10	0,000	0,858	16,960	7	0,115	0,192	0,115	0,154	0,115	0,269
R (RL from R-E)	RICA1a27	0,000	n.d.	n.d.	1	1,000					
R (RL from R-E)	RlCA18	n.d.	n.d.	n.d.	n.d.						
R (RL from R-E)	Rrid135A	0,000	0,719	131,135	5	0,105	0,105	0,263	0,474	0,053	

Notes: LL, *P. lessonae* ; RL_les, L genome from *P. esculentus* ; RR, *P. ridibundus* ;
L (RL from R-E), lessonae genome of P. esculentus from the R-E system; R (RL from R-E), ridibundus genome of P. esculentus from the R-E system;
n.d., not determined; Hobs, heterozygosity observed; Hexp, heterozygosity expexpected; Var, variance in allele length; NA, number of alleles.

0,020		
0,114	0,012	0,223
0,012	0,012	
0,014	0,425	0,173
0,088	0,078	0,044
0,083	0,026	0,029
0,040	0,055	0,025

0,049		
0,110	0,098	0,012
0,088	0,088	0,013
0,560	0,024	0,060
0,066	0,049	0,016
0,026	0,026	
0,197	0,113	0,056

0,136	0,045	0,045
0,045		
0,038		
