# Supplementary figures and images for: All-male hybrids of a tetrapod Pelophylax esculentus share its origin and genetics of maintenance
Source: Biol Sex Differ. 2018 Apr 2;9:13. doi: 10.1186/s13293-018-0172-z (PMC5880063; doi:10.1186/s13293-018-0172-z)

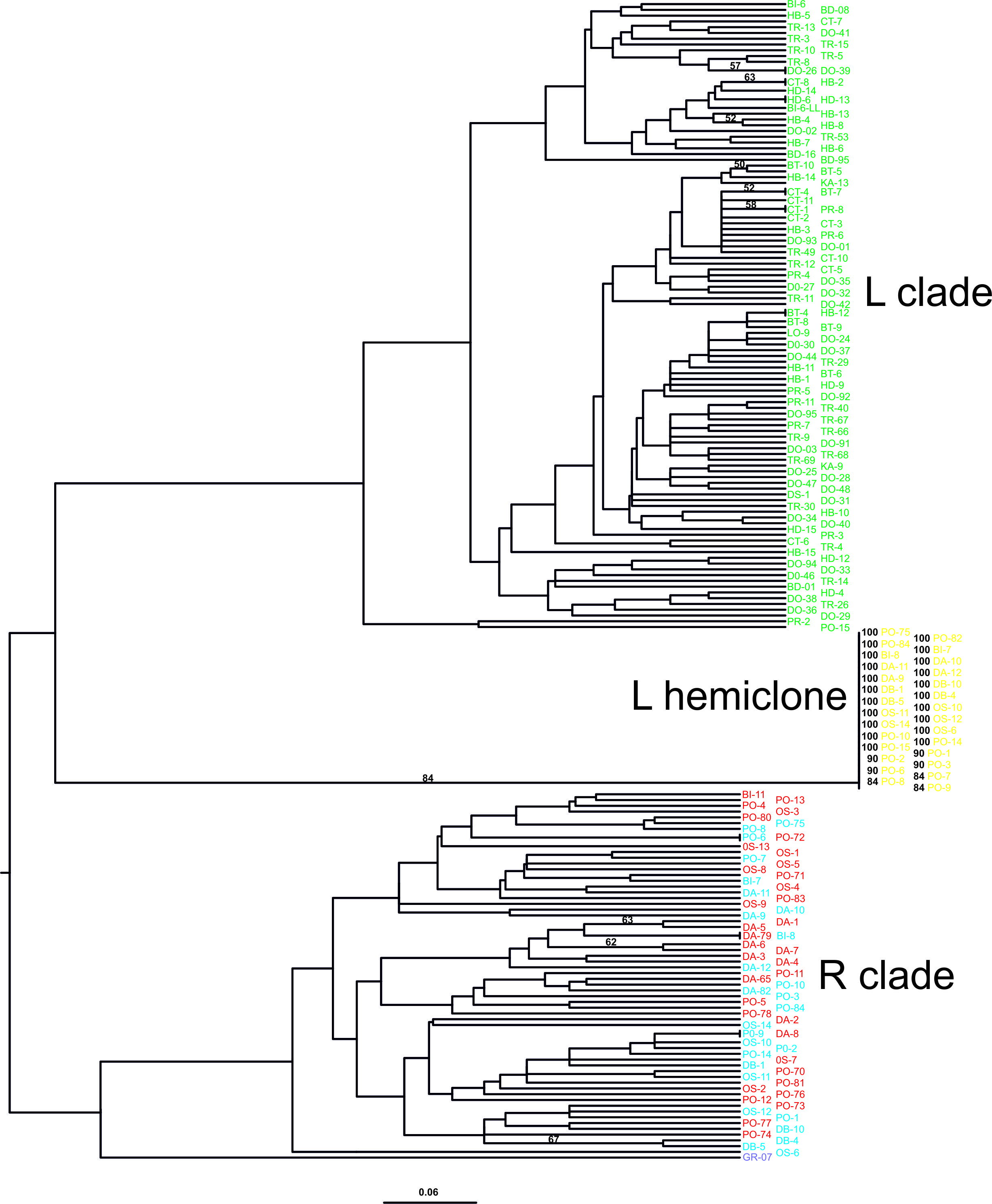

Supplement: Supplementary file 6 — Figure S1: Phylogenetic tree of DA distance of 10 microsatellite loci reconstructed in Populations (method UPGMA, 7 000 replicates, shown only bootstraps above 50 %, distance scale). One terminal branch represents one individual: Green color – P. lessonae, yellow color - L genome from P. esculentus, red colour – P. ridibundus, blue color – R genome from P. esculentus, violet color – P. kurtmuelleri. (JPEG 3388 kb) [file 13293_2018_172_MOESM6_ESM.jpg]
