# Supplementary material for: All-male hybrids of a tetrapod Pelophylax esculentus share its origin and genetics of maintenance
Source: Biol Sex Differ. 2018 Apr 2;9:13. doi: 10.1186/s13293-018-0172-z (PMC5880063; doi:10.1186/s13293-018-0172-z)
Supplement: Supplementary file 7 — Table S6. Multilocus Genotypes (MLGs) derived from P. lessonae, P. ridibundus and P. esculentus from the R-E system based on 17 microsatellite loci. (PDF 316 kb) [file 13293_2018_172_MOESM7_ESM.pdf]

Tab. S6: Multilocus Genotypes (MLGs) derived from *P. lessonae, P. ridibundus* and *P. esculentu* s from the R-E system based on 17 microsatellite loci.

Description: This table lists multilocus genotypes generated by GenAlex v. 6.41 with included P. lessonae, P. ridibundus, lessonae genomes of P. esculentus from the R-E system and ridibundus genomes of P. esculentus from the R-E system.

Sample ID	Multilocus Genotypes
DO-33-LL	07807811311819519525626412112111000014014300000000000001211310000029629627827809709709511118619000000 0g
BD-01-LL	078078118118195195252256121121118124143145000000098110121121000000293296278278097097095111181184000000g
HB-7-LL	078078118118195195252260121121110000145147000000000000119119000000296296278278097097125125177177236236g
DO-38-LL	078078118118195195256256121121110000140145000000000000119131000000296296278278097097095111186190000000g
HB-4-LL	078078118118195195256256121121120000145145000000000000119121000000000000000000097097111111177181000000g
TR-4-LL	078078118118195195256260121121146000145152000000000000123123000000296296278278097097095119177181000000g
CT-6-LL	078078118118195195256262121121108110147154000000000000123123000000296296278278097097111119181186236236g
BI-6-LL	078078118118195195260260121121124000145145000000000000113119000000000000000000097097095111177186000000g
TR-10-LL	078078118118195195260260121121142144145147000000000000115127000000296296278278097097095111181202000000g
DS-2-LL	078078118118195195000000121121116120112145000000000000119119000000000000000000097097095095000000000000g
HB-5-LL	078078118118195195000000121121116120147162000000000000119121000000000000000000097097111119181186000000g
PR-2-LL	078078118134195201252256121121116126140145000000110000098115187000296296278278097106111121179181100099g
DO-46-LL	078080118118195195247256121121110116147162000000000000121131000000296296278278097097119119179186000000g
DO-91-LL	078080118118195195247256121121110000136145000000000000121131000000296296278278097097095111184186000000g
HB-6-LL	078080118118195195252256121121124000145147000000000000119119000000296296000000097097095121177177000000g
TR-15-LL	078080118118195195252260121121124142154154000000000000000000000000000000000000097097095121177181236236g
HD-14-LL	078080118118195195256256121121104110112145000000000000000000000000000000000000000000000000000000000000g
CT-5-LL	078080118118195195256256121121110120147150000000000000117119000000296296278278097097111113177179236236g
TR-12-LL	078080118118195195256256121121110000138145000000000000123127000000296296278278097097095121181186236236g
HD-6-LL	078080118118195195256256121121110000140145000000000000000000000000000000000000000000000000000000000000g
CT-8-LL	078080118118195195256256121121110000145145000000000000119119000000296299278278097097111111177177000000g
DO-35-LL	078080118118195195256256121121110000147154000000000000119131000000296296278278097097119121177195000000g
HD-13-LL	078080118118195195256256121121112120140145000000000000119119000000293296278278097097111121186186236236g
HB-2-LL	078080118118195195256256121121116120145145000000000000119119000000000000000000097097111121181186000000g
DO-27-LL	078080118118195195256256121121116128136145000000000000119125000000296296278278097097111119181186000000g
PR-4-LL	078080118118195195256256121121120122150164000000000000119133187000296296278278097097119121184186236236g
HD-12-LL	078080118118195195256256121121120126140143000000000000119121000000296296278278097097095095186186236236g

DO-94-LL	0780801181181951952562561211211200001361400000000000001211310000002962962782780970971111191791810000000g
DO-02-LL	0780801181181951952562561211211200001451450000000000001191210000002962962782780970971111191861860000000g
TR-53-LL	0780801181181951952562561211210000001451450000000000001231310000002962992782780970970951191861860000000g
HB-8-LL	078080118118195195256258121121108110145145000000000000011912100000029629600000000970970951191811862362 36g
DO-03-LL	0780801181181951952562601211211021101451450000000000001211250000002962962782780970971111191841860000000g
PR-5-LL	078080118118195195256260121121110116147152000000000000011913900000029629627827809709711711917718123623 6g
PR-11-LL	078080118118195195256260121121110120145152000000110000098131227000296296278278097097095095181186236236g
PR-7-LL	078080118118195195256260121121110131147147000000000000011513100000029629627827809709711111918119523623 6g
TR-67-LL	0780801181181951952562601211211100001121450000000000001151310000002962962782780970971111111811900000000g
TR-66-LL	0780801181181951952562601211211121141361470000000000001211310000002962962782780970970950951811810000000g
TR-69-LL	0780801181181951952562601211211200001451470000000000001231250000002962962782780970970951191811810000000g
DO-95-LL	0780801181181951952562601211211200001451620000000000001271310000002962962782780970971111211791810000000g
TR-40-LL	0780801181181951952562601211211421441451520000000000001191310000002962962782780970971191271811860000000g
TR-9-LL	078080118118195195256260121121142000150152000000000000012712700000029629627827809709711311917718123623 6g
DO-92-LL	0780801181181951952562601520001160001451620000000000001191210000002962962782780970971111151861900000000g
DO-32-LL	0780801181181951952562641211211100001361470000000000001191250000002962962782780970971191271861900000000g
BT-6-LL	078080118118195195256264121121128000145150000000000000011912100000029629627827809709711111917718123623 6g
BD-16-LL	0780801181181951952582601211211221241451450000000000001191190000002962962782780970970951111861860000000g
CT-7-LL	0780801181181951952602601211211081101121300000000000001171191910002962962782780970971111131771810000000g
DO-26-LL	0780801181181951952602601211211101141451470000000000001311310000002962962782780970971191211861900000000g
DO-41-LL	0780801181181951952602601211211101141501520000000000001191210000002962962782780970971191211751810000000g
TR-13-LL	078080118118195195260260121121110120145152000000000000011912300000029629627827809709711912117720223623 6g
BD-08-LL	0780801181181951952602601211211101241451450000000000001291290000002932872782780970970951191861860000000g
TR-3-LL	078080118118195195260260121121110146152154231000110000115119191227296296278278097106095119181181236236g
TR-68-LL	0780801181181951952602601211211100001451450000000000001211310000002962962782780970970951191811860000000g
DO-39-LL	0780801181181951952602601211211160001451470000000000001311310000002962962782780970971191211861950000000g
TR-5-LL	078080118118195195260260121121120000140147000000110000127131000000296296278278097097095095181186236236g
TR-8-LL	078080118118195195260260121121120000145147000000110000127131191000296296278278097097095095181186236236g
HB-13-LL	0780800000000000000000001211211200001451450000000831101191290000000000000000000000970971111111881880000000g
DO-36-LL	0780831131181951952562601211211101241401450000000000001231310000002962962782780000001111271771860000000g
HD-4-LL	078083118118195195256256121121110120145152000000110000119131187000296296278278097097119125186188236236g
TR-26-LL	0780831181181951952562601211211121201451500000000000001251310000002962962782780970971111191811950000000g
DO-29-LL	0780831181181951952562600000001101241401400000000000001311310000002962992782780970970951271751790000000g

HB-1-LL	080080118118195195252256121121120000140145000000000000121129000000296296278278097097111121177188000000g
BT-8-LL	080080118118195195252260121121116126145147000000000000119121000000296296278278097097095111181181000000g
DO-01-LL	080080118118195195256256121121102110145145000000000000121121000000296296278278097097111121181186000000g
CT-2-LL	080080118118195195256256121121108116145147000000000000131131000000296296278278097097111111177186236236g
HB-3-LL	080080118118195195256256121121110116145145000000000000125131000000296296278278097097111111177188236236g
BT-5-LL	080080118118195195256256121121110116145147000000000000119119000000296296278278097097111111177181000000g
PR-8-LL	080080118118195195256256121121110116147150000000000000000000000000000000000000000000000000000000000000g
PR-6-LL	080080118118195195256256121121110120140145000000000000125125000000296299278278097097111119179188236236g
Ka-13-LL	080080118118195195256256121121110120147156000000000000000000000000000000000000000000000000000000000000g
PR-3-LL	080080118118195195256256121121110000123147000000110000098125187227291296278278097116095115181186236236g
DO-93-LL	080080118118195195256256121121110000138150000000000000123127000000293296278278097097111111175190000000g
CT-1-LL	080080118118195195256256121121116000147150000000000000113131000000000000000000097097111111186186000000g
BT-10-LL	080080118118195195256256121121120126145147000000000000119127000000296296278278097097095111177181236236g
CT-3-LL	080080118118195195256256121121120142145147000000000000123131000000293299278278097097111119186186000000g
CT-11-LL	080080118118195195256256121121120000145152000000000000117121000000293296278278097097111111181181000000g
BT-7-LL	080080118118195195256256121121126000145150000000000000000000000000000000000000000000000000000000000000g
TR-49-LL	080080118118195195256256121121000000145152000000000000119131000000296296278278097097095095181186000000g
Ka-9-LL	080080118118195195256260121121108110136145000000000000119131000000296299278278097097095119181197236236g
LO-9-LL	080080118118195195256260121121108110140145000000110000119125191234296296278278097097095111184184000000g
DO-44-LL	080080118118195195256260121121110124140147000000000000119131000000296296278278097097095119181186000000g
DO-31-LL	080080118118195195256260121121110124140162000000000000119131000000296296278278097097111111184188000000g
DO-25-LL	080080118118195195256260121121110124145145000000000000125131000000296299278278097097119127184186000000g
DO-30-LL	080080118118195195256260121121110128145152000000000000119125000000296296278278097097111111181188000000g
TR-29-LL	080080118118195195256260121121110000147150000000000000121123000000296296278278097097095095181181000000g
BT-9-LL	080080118118195195256260121121116126145147000000000000121121000000296296278278097097095111181181000000g
DO-48-LL	080080118118195195256260121121120124136136000000000000125131000000296296278278097097113119181186000000g
BT-4-LL	080080118118195195256260121121120126145147000000000000119121000000296296278278097097111125177181000000g
HD-9-LL	080080118118195195256260121121120000140152000000000000113121000000296296278278097097095111181186236236g
DO-24-LL	080080118118195195256260121121128000140145000000000000119123000000296296278278097097095119177190000000g
CT-10-LL	080080118118195195256262121121120000138147000000110000119119000000296296278278097097111119177186236236g
DO-47-LL	080080118118195195256264121121110000136145000000000000125131000000296296278278097097111127184190000000g
TR-30-LL	080080118118195195260260121121110000145150000000000000131131000000296296278278097097111111177181000000g
HB-11-LL	080080118118195195260260121121116126145147000000000000119131000000000000000000097097111111177186000000g

DO-28-LL	080080118118195195260260121121122128145145000000000000119123000000296299278278097097111119190195000000g
BD-95-LL	080080118118195195260260121133118000112145000000000000119121000000293299278278097097119125181184000000g
DS-1-LL	080080118118195195260262121121120000112138000000000000119123000000296296278278097097095119181186000000g
CT-4-LL	080080118118195195000000121121110120145150000000000000113121000000000000278278097097111119177181000000g
HB-12-LL	080080118118195195000000000000000000000000000000000000119121000000000000000000097097095125181181000000g
HB-14-LL	080080000000195195256256121121110120145147000000000000119121000000000000000000097097095110000000000009g
TR-11-LL	080083118118195195256256121121110124145145000000000000123135000000296296278278097097095095184184236236g
HB-10-LL	080083118118195195256256121121120126145152000000110000121125000000000000000000097097095111177184000000g
DO-34-LL	080083118118195195256256121121128000145147000000000000121131000000296296278278097097095111179186000000g
DO-40-LL	080083118118195195256256000000116128145147000000000000121121000000296296278278097097095115186190000000g
TR-14-LL	080083118118195195256260121121110120112140235000000000131141000000296296278278097116095115181181100099g
DO-37-LL	080083118118195195256260121121116000145147000000000000121123000000296296278278097097111111184184000000g
HD-15-LL	080083118118195195256260121121120124145152000000000000119119000000291296278278097097111121177181236236g
DO-42-LL	080083118118195195256260121121120000145150000000000000115123000000296296278278097097119121177177000000g
HB-15-LL	080083118134195195000000121121120000140145000000110000125131000000000000000000097097119110000000000009g
PO-1-L	078078118118195195260260121121120120145145000000000000123123000000296296278278097097115115186186236236g
PO-2-L	078078118118195195260260121121120120145145000000000000123123000000296296278278097097115115186186000000g
PO-82-L	078078118118195195260260121121120120145145000000000000123123000000296296278278000000115115186186000000g
PO-9-L	078078118118195195260260121121120120145145000000000000123123000000000000000000097097115115186186000000g
PO-3-L	078078118118195195000000121121120120145145000000000000123123000000000000000000097097115115186186000000g
PO-8-L	078078118118195195260260121121120120145145000000000000123123000000296296000000097097115115186186000000g
PO-7-L	078078118118000000000000121121120120145145000000000000123123000000000000000000097097115115186186000000g
PO-15-L	078078000000195195000000121121120120145145000000000000123123000000000000000000097097115115186186000000g
DB-8-RR	074092134134201243232000115000000000106110169220110110098098187000281287311313106108000000000000169199g
OS-3-RR	078092134134201253232000127000000000106106169200110116098098195214281281313317106000000000000000203203g
OS-8-RR	078092134134205253232000115117000000106106231255110110098098187198281281303315114116000000000000199203g
OS-13-RR	083085134136201217234236115127000000106106204208110129098098189191281287313313106000000000000000169203g
PO-83-RR	083092134134201205000000115000000000106106169192110116098098212000281287313317108000000000000000205205g
PO-4-RR	083092134134201217000000127000000000106106169192104110098098180000000000000000091061220000000000203203g
OS-1-RR	083092134134205209232000127000000000106106169200083083098098191198281281000000108116000000000000199203g
DB-9-RR	085085134134201243232250127000000000106106169231110110098098191207281281311313106114000000000000169203g
PO-77-RR	085085134134205223000000000121000000106106169231110124098098191000281287311313108116000000000000199199g
Da-8-RR	085092134134201201000000127000000000106110169235110110098098187195281281313317106000000000000000199203g

OS-2-RR	08509213413420120523400012700000000011011020020811012000000918720328128731332111600000000000000169203g
Da-4-RR	08509213413420124723226011712300000010610619221208712909809818700028729630731310611400000000000000169169g
Da-5-RR	08509213413420124723200011912300000010610619221208312909809818700028128130731309310600000000000000169169g
Da-1-RR	08509213413420124723200011912300000010610621223108712909809818719128128131331310611400000000000000169169g
DB-3-RR	08509213413420520523224812700000000000000169169110110098098191203281281313317106108000000000000000169203g
PO-73-RR	08509213413420521700000000121000000106106169169114116098098191000281287313315106000000000000000203203g
PO-81-RR	0850921341342172400000091270000000001101101691691101100980981910002812813173171160000000000000000199203g
OS-9-RR	085092134136201205232234127000000000106106212235083110098098191000287287307313112118000000000000000000g
PO-72-RR	085094132134223223000000127000000000106106208220098108000000191000281287307313114118000000000000000199205g
PO-76-RR	0850981341342432430000001270000000001061101691691041100980982030002812813133191140000000000000000199199g
PO-80-RR	0920921341342012010000001270000000001061061692311101100980981910002812873113131081060000000000000199203g
OS-7-RR	0920921341342012052322501270000000001061102002200831100980981870002812813153171081160000000000000199205g
PO-79-RR	09209213413420120523200000012100000010610619220011011609809819121228128130731310611400000000000000203203g
OS-4-RR	0920921341342012052340001150000000001061062122201101100980981912032812813153210971160000000000000169199g
Da-3-RR	09209213413420124723200011712300000010610621223108711009809818700028729631331511400000000000000000169199g
Da-7-RR	09209213413420124723200011000900000010610616923108312909809818719128129630731309310600000000000000169203g
Da-6-RR	09209213413420124723200011000900000010610616923108312909809818700028729631331309310600000000000000169203g
PO-5-RR	09209213413420520523200000012100000011011016920008310809809819120728128131113131180000000000000000199203g
PO-71-RR	0920921341342052170000001270000000001061061691691101100980982120002812813133131081060000000000000203203g
DB-6-RR	092092134134243243232260127000000000106106169227110110098098191000281281311131311413700000000000000203203g
PO-70-RR	0920921341362012010000000012100000010611016923511011609809819520728128131333131116122000000000000000203203g
DB-2-RR	092092134138201243232248000121000000106106169196110110098098191203275281313317110114000000000000000169203g
PO-65-RR	09209413413420124323200000012100000010611016916911011009809820700028128731333131180000000000000000203203g
BI-11-RR	092094134134201259234000127000000000106106220235110110098098187000281281000000106000000000000000000169199g
PO-12-RR	0920941341342392472320001270000000001061100000001061100980981892030000003133131251400000000000000000000g
PO-13-RR	09209813413420120100000012700000000010610616922011012709809818719528128131113131061370000000000000199199g
OS-5-RR	092098134134201255232000127000000000106106220235106110098098187198281281303313112114000000000000000199203g
Da-2-RR	0920981341342052052322501270000000001061102082350831100980981910002812873033131110000000000000000169183g
PO-11-RR	09409413413420124323200000012100000010611020023111011000000019100028128131333191081140000000000000169203g
PO-78-RR	0940941341342052050000000110009000000110110169192110114098098000000281281313331310811400000000000000169169g
PO-74-RR	09409413413420524700000000012100000010610621223108310609809819120328128731333131081100000000000000199203g
DB-7-RR	000001341340000000000000000000000000000000011011009809818719128128131331511413700000000000000000000g
OS-6-R	0830831341342012010000000115115000000106106200200110110098098214214000000319319116116000000000000000000000g

PO-15-R	083083000000000000000000000000000000000176176110110098098000000000000000000000112112000000000000000000g
PO-9-R	085085134134201201000000127127000000106106169169110110098098203203000000000000106106000000000000203203g
OS-12-R	085085134134201201000000000000000000106106196196110110098098191191287287315315116116000000000000000000g
Da-11-R	085085134134205205234234127127000000106106208208133133098098198198281281315315116116000000000000199199g
DB-10-R	085085134134205205000000127127000000106106208208110110098098189189287287319319108108000000000000203203g
PO-1-R	085085134134209200000000000000090000001061061691691101100980982032032812813153151121120000000000000169169g
PO-3-R	092092132132201201000000000000000000110110000000110110098098000000000000000000108108000000000000000000g
Da-10-R	092092134134201201232232127127000000000000212212083083000000198198281281311311112112000000000000183183g
Da-9-R	092092134134201201000000115115000000000000212212083083098098198198281281311311112112000000000000183183g
DB-1-R	092092134134201201000000127127000000110110235235110110098098179179281281321321110110000000000000199199g
PO-75-R	092092134134201201000000127127000000000000196196083083098098187187281281313313108108000000000000203203g
PO-2-R	092092134134201201000000129129000000110110000000110110098098187187281281313313116116000000000000199199g
OS-11-R	092092134134201201000000000000000000106106196196110110098098191191281281315315116116000000000000000000g
BI-8-R	092092134134201201000000000000000000106106000000110110098098191191000000000000106106095095000000000000g
OS-10-R	092092134134205205236236127127000000110110212212110110098098198198281281313313116116000000000000199199g
BI-7-R	092092134134205205256256127127000000000000000000110110098098187187000000000000106106000000000000000000g
PO-84-R	092092134134205205000000000000000000110110204204098098000000191191281281313313114114000000000000203203g
DB-5-R	092092134134223223000000000000000000000000169169110110098098191191281281313313110110000000000000169169g
PO-14-R	092092134134243243000000127127000000106106000000104104098098000000000000000000116116000000000000000000g
DB-4-R	092092134134243243000000000000000000000000169169110110098098191191281281313313110110000000000000205205g
Da-12-R	092092134134247247232232115115000000106106231231083083000000187187281281000000114114000000000000203203g
OS-14-R	092092136136205205000000127127000000110110169169104104000000191191281281313313114114000000000000203203g
PO-7-R	092092000000000000000000127127000000106106169169110110098098000000000000000000108108000000000000203203g
PO-8-R	094094134134201201000000127127000000106106169169110110098098191191287287000000108108000000000000203203g
PO-10-R	094094134134201201000000000000000000110110169169110110098098000000281281313313000000000000000000199199g
PO-82-R	094094134134223223000000000000000000106106169169110110098098191191281281313313122122000000000000203203g

Notes: LL, *P. lessonae* ; RR, *P. ridibundus;* Hybrid L, *lessonae* genome of *P. esculentus* from the R-E system; Hybrid R, *ridibundus* genome of P. esculentus from the R-E system.

MLG´s ID	Type of MLG	No. of ind.
1	LL	1
2	LL	1
3	LL	1
4	LL	1
5	LL	1
6	LL	1
7	LL	1
8	LL	1
9	LL	1
10	LL	1
11	LL	1
12	LL	1
13	LL	1
14	LL	1
15	LL	1
16	LL	1
17	LL	1
18	LL	1
19	LL	1
20	LL	1
21	LL	1
22	LL	1
23	LL	1
24	LL	1
25	LL	1
26	LL	1
27	LL	1

28	LL	1
29	LL	1
30	LL	1
31	LL	1
32	LL	1
33	LL	1
34	LL	1
35	LL	1
36	LL	1
37	LL	1
38	LL	1
39	LL	1
40	LL	1
41	LL	1
42	LL	1
43	LL	1
44	LL	1
45	LL	1
46	LL	1
47	LL	1
48	LL	1
49	LL	1
50	LL	1
51	LL	1
52	LL	1
53	LL	1
54	LL	1
55	LL	1
56	LL	1
57	LL	1
58	LL	1
59	LL	1
60	LL	1

61	LL	1
62	LL	1
63	LL	1
64	LL	1
65	LL	1
66	LL	1
67	LL	1
68	LL	1
69	LL	1
70	LL	1
71	LL	1
72	LL	1
73	LL	1
74	LL	1
75	LL	1
76	LL	1
77	LL	1
78	LL	1
79	LL	1
80	LL	1
81	LL	1
82	LL	1
83	LL	1
84	LL	1
85	LL	1
86	LL	1
87	LL	1
88	LL	1
89	LL	1
90	LL	1
91	LL	1
92	LL	1
93	LL	1

94	LL	1
95	LL	1
96	LL	1
97	LL	1
98	LL	1
99	LL	1
100	LL	1
101	LL	1
102	LL	1
103	LL	1
104	LL	1
105	LL	1
106	LL	1
107	LL	1
108	LL	1
109	Hybrid L	14
110	Hybrid L	3
111	Hybrid L	2
112	Hybrid L	3
113	Hybrid L	2
114	Hybrid L	1
115	Hybrid L	1
116	Hybrid L	1
117	RR	1
118	RR	1
119	RR	1
120	RR	1
121	RR	1
122	RR	1
123	RR	1
124	RR	1
125	RR	1
126	RR	1

127	RR	1
128	RR	1
129	RR	1
130	RR	1
131	RR	1
132	RR	1
133	RR	1
134	RR	1
135	RR	1
136	RR	1
137	RR	1
138	RR	1
139	RR	1
140	RR	1
141	RR	1
142	RR	1
143	RR	1
144	RR	1
145	RR	1
146	RR	1
147	RR	1
148	RR	1
149	RR	1
150	RR	1
151	RR	1
152	RR	1
153	RR	1
154	RR	1
155	RR	1
156	RR	1
157	RR	1
158	RR	1
159	Hybrid R	1

160	Hybrid R	1
161	Hybrid R	1
162	Hybrid R	1
163	Hybrid R	1
164	Hybrid R	1
165	Hybrid R	1
166	Hybrid R	1
167	Hybrid R	1
168	Hybrid R	1
169	Hybrid R	1
170	Hybrid R	1
171	Hybrid R	1
172	Hybrid R	1
173	Hybrid R	1
174	Hybrid R	1
175	Hybrid R	1
176	Hybrid R	1
177	Hybrid R	1
178	Hybrid R	1
179	Hybrid R	1
180	Hybrid R	1
181	Hybrid R	1
182	Hybrid R	1
183	Hybrid R	1
184	Hybrid R	1
185	Hybrid R	1
